# Differences in the Establishment of Gut Microbiota and Metabolome Characteristics Between Balb/c and C57BL/6J Mice After Proton Irradiation

**DOI:** 10.3389/fmicb.2022.874702

**Published:** 2022-05-06

**Authors:** Yuchen Li, Li Sui, Hongling Zhao, Wen Zhang, Lei Gao, Weixiang Hu, Man Song, Xiaochang Liu, Fuquan Kong, Yihao Gong, Qiaojuan Wang, Hua Guan, Pingkun Zhou

**Affiliations:** ^1^Hengyang Medical School, University of South China, Hengyang, China; ^2^Beijing Institute of Radiation Medicine, Beijing, China; ^3^Department of Nuclear Physics, China Institute of Atomic Energy, Beijing, China; ^4^College of Life Sciences, Hebei University, Baoding, China

**Keywords:** proton irradiation, gut microbiota, intestinal injury, different strain mice, metabolism

## Abstract

Although proton irradiation is ubiquitous in outer space as well as in the treatment of human diseases, its effects remain largely unclear. This work aimed to investigate and compare the composition of gut microbiota composition of mice in different species exposed to high-dose radiation. Male Balb/c mice and C57BL/6J mice were irradiated at a high dose (5Gy). Fecal specimens before and after irradiation were subjected to high-throughput sequencing (HTS) for the amplification of 16S rRNA gene sequences. We observed substantial changes in gut microbial composition among mice irradiated at high doses compared to non-irradiated controls. The changes included both the alpha and beta diversities. Furthermore, there were 11 distinct alterations in the irradiation group compared to the non-radiation control, including the families Muribaculaceae, Ruminococcaceae, Lactobacillus, Lachnospiraceae_NK4A136, Bacteroides, Alistipes, Clostridiales, Muribaculum, and Alloprevotella. Such alterations in the gut microbiome were accompanied by alterations in metabolite abundances, while at the metabolic level, 32 metabolites were likely to be potential biomarkers. Some alterations may have a positive effect on the repair of intestinal damage. Simultaneously, metabolites were predicted to involve multiple signal pathways, such as Urea Cycle, Ammonia Recycling, Alpha Linolenic Acid and Linoleic Acid Metabolism, Ketone Body Metabolism, Aspartate Metabolism, Phenylacetate Metabolism, Malate-Aspartate Shuttle, Arginine and Proline Metabolism and Carnitine Synthesis. Metabolites produced by proton irradiation in the microbial region play a positive role in repairing damage, making this area worthy of further experimental exploration. The present work offers an analytical and theoretical foundation to investigate how proton radiation affects the treatment of human diseases and identifies potential biomarkers to address the adverse effects of radiation.

**Importance:**

The space radiation environment is extremely complex, protons radiation is still the main component of space radiation and play an important role in space radiation. We proposed for the first time to compare the feces of Balb/c and C57BL/6J mice to study the changes of intestinal flora before and after proton irradiation. However, the effect of proton irradiation on the gut microbiome of both types of mice has not been previously demonstrated. After proton irradiation in two kinds of mice, we found that the characteristics of intestinal microbiome were related to the repair of intestinal injury, and some metabolites played a positive role in the repair of intestinal injury.

## Introduction

Astronauts on missions into outer space outside the low Earth orbit are exposed to space radiation (Suman et al., 2016). The Sun usually emits solar energetic particle events (SPE), which pose a risk to human health during the process of the 11-year solar cycle. The SPE is mainly in the form of protons (Townsend, 2005; Cengel et al., 2010). Recently, proton radiotherapy has been increasingly used in human patients, and corresponding research has also been conducted on animals (Slater, 2006; Choi et al., 2019; Suckert et al., 2020). Radiotherapy of head and neck cancer (HNC) is the final therapeutic strategy or a postoperative adjuvant therapy (Pfister et al., 2020). A study by Chang proves that proton radiotherapy provides better local control in non-small cell lung cancer (NSCLC) and meningiomas (Chang et al., 2017). The study by Lin et al. (2020) has demonstrated that proton radiotherapy reduces the risk and severity of advanced esophageal cancer. Another study by Holtzman et al. (2019) has revealed that skull-base chondrosarcoma can be effectively treated either by single proton therapy or that after surgery. Nonetheless, although radiation has an excellent capability of killing tumor cells, it can also cause certain adverse reactions. For instance, it cannot fully avoid damage to surrounding tissues during irradiation at the tumor site.

The intestine is particularly sensitive to ionizing radiation, resulting in side effects including vomiting, weight loss, anorexia, dehydration, diarrhea, and infections (Dupree et al., 2000; Preston et al., 2003, 2007; Endo et al., 2011; Mutoh et al., 2011; Kumar et al., 2018). The intestinal epithelium represents an excellent model for the study of tissue regeneration and homeostasis following radiation injury owing to its self-renewing capacity (Choi et al., 2019). The relationship between microbes and health has long been a topic of interest, but our understanding of the relationship between gut microbes and health has been minimal for a long time. In recent years, with the continuous development and application of new research methods such as high-throughput sequencing, the impact of gut microbes on human health has regained attention and become a current research hotspot in life sciences and medicine. Several articles have emphasized that gut microbiota play a dual role in the preservation of host health. Gut microbiota represents the heterogeneous commensal microbial populations, including bacteria, fungi, viruses, and archaea, which are colonized in the intestine, mainly in the large intestine. We are usually exposed to high doses of radiation in our life (Lynch and Pedersen, 2016). Gut microbiota is responsible for numerous critical functions, which include the production of vitamins, the metabolism of dietary compounds, and the protection of the gut from the infiltration and spread of pathogens (Vaishnava et al., 2008; Belkaid and Naik, 2013; Magnúsdóttir et al., 2015). The balance of gut microbiota is important for fulfilling such critical metabolic functions. When we study gut microbes, we often collect mouse feces as the object of analysis (Li et al., 2020a,b; Xiao et al., 2020). However, a study by Bannister has demonstrated that the diverse murine strains, BALB/c and C57BL/6, have different radio sensitivities to diverse doses of radiation (Bannister et al., 2016). Also, BALB/c mice, but not C57BL/6 mice, are sensitive to radiation-induced lethality (Rivina et al., 2016). Considering such a discrepancy in the existing articles, this work aimed to investigate the complicated relationship between different strains and microbial metabolism during radiation intestinal injury (RII), and particularly how the detrimental and protective bacterial metabolites affected injury and inflammation. Hopefully, the present work has contributed to the systemic and comprehensive interrogation of the metabolome and microbiome of mouse RII fecal samples for identifying the metabolite levels and microbial diversity, as well as exploring the relationships between RII, fecal metabolites, and gut microbiota. Rather than the singular pathogenic microorganism, this study suggested that the microbial net metabolic output, as well as the elicited inflammatory signals, made major contributions to RII, which must be investigated in future studies.

## Results

### The Decreased Fecal Bacterial Diversity Was Related to Proton Irradiation

**FIGURE 1:**
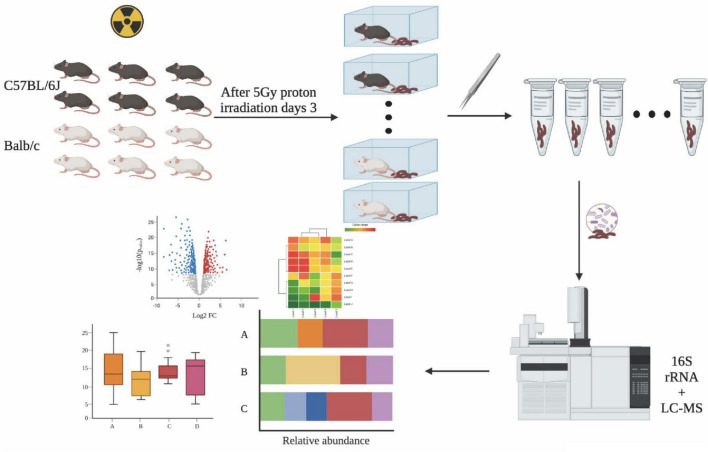
Experimental procedure. Fecal samples were collected from irradiated mice and processed for 16S rRNA amplicon and LC-MS profiling. This image was created with BioRender (https://biorender.com/).

**FIGURE 2:**
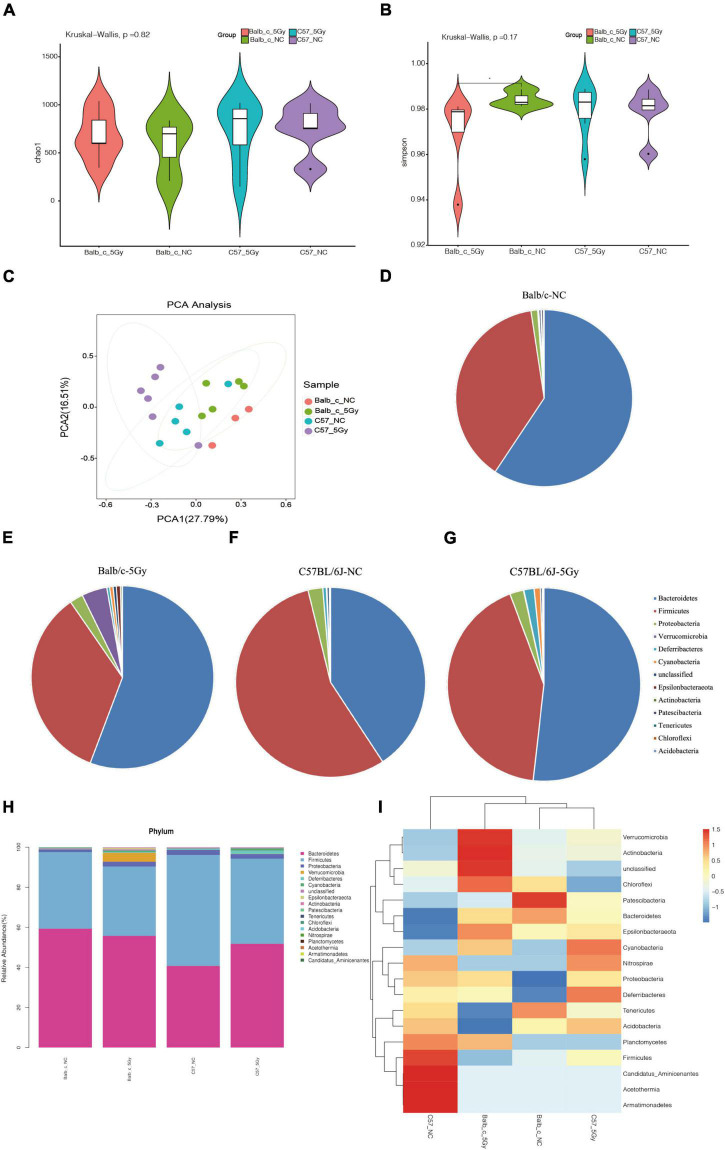
Changes of gut microbiota after proton irradiation in each group at phylum level. **(A)** The Alpha diversity assessed by richness (Chao1) and **(B)** evenness diversity (Simpson) of the intestinal bacteria in male mice at days 3 post-proton irradiation were examined by 16S high-throughput sequencing. (Wilcoxon, *p < 0.05). **(C)** Principal Component Analysis (PCA) score plots based on Bray–Curtis distance at phylum level. Average abundance of bacterial phyla in the Balb/c-NC **(D)**, Balb/c-5Gy **(E)**, C57BL/6J-NC **(F)**, and C57BL/6J-5Gy **(G)** mice intestinal microbiota. **(H)** Taxonomic summary of the gut microbiota of Balb/c and C57BL/6J at phylum level. **(I)** Heatmap analysis of gut microbiota changes from different mice group at phylum level. (Mean ± SD; Balb/c-NC: *n* = 3; Balb/c-5Gy: *n* = 6; C57BL/6J-NC: *n* = 6; C57BL/6J-5Gy: *n* = 6).

To investigate the changes in gut microbial composition in the fecal samples from different strains after exposure to proton irradiation, we obtained fecal samples from mice on day three following 5Gy proton irradiation (Figure 1). The bacterial composition in the fecal samples was determined using the 16S rRNA amplicon sequencing data. Then, the gut microbial compositions were compared between diverse groups through the detection of the bacterial α- and β-diversities. The α-diversity, detected through four indices, namely, Observed, Chao1, Simpson, and Shannon, did not change significantly between the two groups. In contrast, in the Balb/c strain, the community diversity measured through the Simpson index was remarkably reduced in the proton irradiation group compared to the normal control (NC) group (*p* = 0.04, Wilcoxon sum test) (Figures 2A–C, also see Supplementary Figure 1).

### Alterations in Gut Microbial Populations in Mouse Feces After Proton Irradiation

It was observed that C57BL/6J and Balb/c mice had different intestinal microbial compositions. Although the degree of difference varied, they were still included in the subsequent analysis. To explore the bacterial taxa associated with Balb/c and C57BL/6J, differential abundance analyses were conducted at both phylum and genus levels in both groups.

At the phylum level, Principal Components Analysis (PCA) between the four groups showed a clear distinction of Balb/c-5Gy and C57BL/6J-5Gy mice from their corresponding NC group (Figure 2C). To identify the similarities of gut microbiota between the samples of the Balb/c-NC, Balb/c-5Gy, C57BL/6J-NC, C57BL/6J-5Gy groups, Bray–Curtis distance was performed based on OTU abundance and presented as a hierarchical clustering tree using the data. The results showed that the samples from each group could be grouped into four clusters, including the Balb/c-NC, Balb/c-5Gy, C57BL/6J-NC, C57BL/6J-5Gy clusters. In these clusters, the unweighted Unifrac distances indicate that C57BL/6J-NC, C57BL/6J-5Gy is divided, while the weighted Unifrac distances indicate that the samples are divided into four groups, especially clearly in the Balb/c-NC, Balb/c-5Gy groups (Supplementary Figures 1D,F).

Microbial taxa were assigned to four groups to assess the relative abundances of predominant taxa at the phylum level. It was observed that the gut microbial species varied significantly between different samples of the same group. The relative abundance of the top ten bacterial phyla among the different strain mice groups is shown in Figures 2D–G (also see Supplementary Table 1). The dominant bacteria in all samples were *Bacteroides*, followed by *Firmicutes*, *Proteobacteria*, *Verrucobacteria*, *Deferrobacterium*, *Cyanobacteria*, *Epsilonbacteraeota*, and *Actinobacteria*, *Patescibacteria, Tenericutes* among others (Figure 2H).

The differential abundance analysis was then conducted using the Mann-Whitney U test, an approach that uses a rank-sum test to accurate exploration of bacterial taxa associated with the different gut microbiota compositions of the C57BL/6J and Balb/c groups (Supplementary Table 2). We obtained the relative abundance values by normalizing the microbiome abundance and comparing them. The abundance of each grouping is the average of all biological replicates within that group. Then, we plotted one heatmap based on the hierarchical cluster analysis using 18 most significantly different taxa to summarize the alterations between samples of the Balb/c and C57BL/6J groups (Figure 2I). The gut microbiota showed significant differences between the Balb/c and C57BL/6J samples (Figure 2I). Among these bacteria, *Patescibacteria, Bacteroidetes* in Balb/c-NC, *Verrucomicrobia, Actinobacteria, Chloroflexi* in Balb/c-5Gy, *Firmicutes, Candidatus_Aminicenantes, Acetothermia, Armatimonadetes* in C57 BL/6J-NC, *Deferribacteres, Cyanobacteria* in C57 BL/6J-5Gy varied significantly between the C57BL/6J and Balb/c groups, while the composition of the other bacterial communities was slightly different and not significantly. We hypothesize that the variation in these colonies is partly due to differences in mouse species and partly due to post-proton irradiation.

**FIGURE 3:**
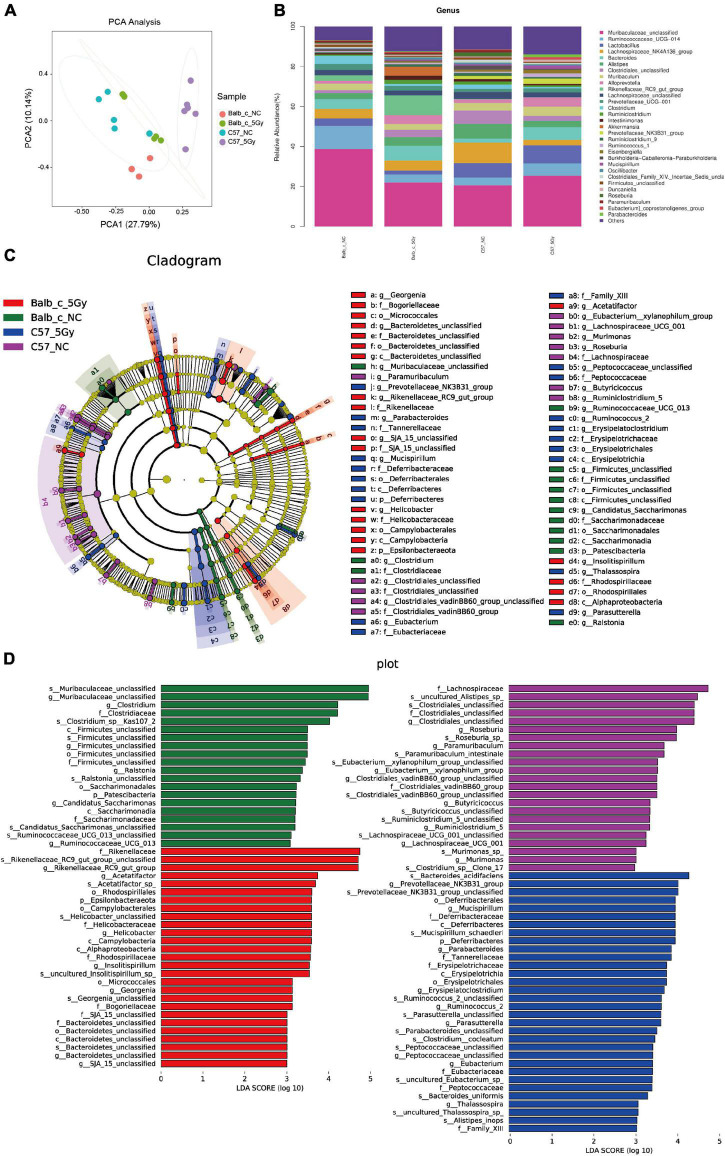
Cladogram and linear discriminant analysis (LDA) by LEfSe analysis showing the biomarker taxa associated with Balb/c and C57BL/6J group. **(A)** PCA score plots based on Bray–Curtis distance at genus level. **(B)** Taxonomic summary of the gut microbiota of Balb/c and C57BL/6J at genus level. **(C)** A cladogram using the linear discriminant analysis (LDA) effect size (LEfSe) analysis shows the phylogenetic distribution of the different groups of gut microbiota. Each successive circle represents a phylogenetic level. Dot size is proportional to the abundance of the taxon. [phylum (p), class (c), order (o), family (f), genera (g), species (s)]. **(D)** Histogram of the LDA scores reveals the most differentially abundant taxa among different treatments. (LDA > 3, *P* < 0.05; Balb/c-NC: *n* = 3; Balb/c-5Gy: *n* = 6; C57BL/6J-NC: *n* = 6; C57BL/6J-5Gy: *n* = 6).

At the genus level, PCA of the four groups that Balb/c-5Gy and C57BL/6J-5Gy mouse are separated from the corresponding non-irradiation group (Figure 3A). Similarly, as observed in the phylum level, there was a significant difference in gut microbiota among samples from every group at the genus level. Over thirty genera were identified among the groups. The dominant bacteria in all samples were *Bacteroidetes, Muribaculaceae*, followed by *Ruminococcaceae, Lactobacillus, Lachnospiraceae_NK4A136_group, Bacteroides, Alistipes, Clostridiales, Muribaculum, and Alloprevotella*, among others (Figure 3B). Among the top 10, *Alistipes, Clostridiales, Lachnospiraceae_NK4A136_group*, and *Rikenellaceae_RC9_gut_group* showed significant differences between Balb/c mice and C57BL/6J mice, while the composition of other bacterial communities showed slight and non-significant differences (also see Supplementary Figures 2A–D).

Taxa with differential abundances were also analyzed using LEfSe (Segata et al., 2011). Comparison of mice in the Balb/c-NC, Balb/c-5Gy, C57BL/6J-NC and C57BL/6J-5Gy groups revealed 18 phylum-level classes of microbiota and 32 genus-level microbiotas, showing significant differences (Figures 3C,D). Consistent with the analysis of differences in microbiota at the phylum level, there were also significant differences in microbiota at the genus level (Supplementary Figure 2E).

As suggested by the results of the linear discriminant analysis (LDA) (Figure 3D), we adopted the LEfSe analysis to statistically compare the Balb/c-NC, Balb/c-5Gy, C57BL/6J-NC, and C57BL/6J-5Gy groups to better understand the differential bacterial community abundances of each group. It was observed that proton irradiation affected the gut microbial composition at different taxonomic levels. There were significant differences in the microbial composition between the Balb/c-NC and Balb/c-5Gy groups. Compared to the Balb/c-NC group, the abundances of *Rikenellaceae*, *Rikenellaceae_RC9_gut_group*, and *Rikenellaceae_RC9_gut_group_unclassified* belonging to the phylum *Chloroflexi* were enhanced in the Balb/c-5Gy group [LDA scores (log10) > 3]. Moreover, the abundance of *Muribaculaceae, Clostridiaceae*, and *Clostridium* was enhanced in the Balb/c-NC mice (Figure 3D, also see Supplementary Figure 3A).

Compared to the C57BL/6J-NC mice, *Bacteroidaceae, Bacteroides, and Bacteroides_acidfaciens* belonging to phylum Bacteroidetes showed higher abundances in the C57BL/6J-5Gy mice [LDA scores (log10) > 3]. Besides, the abundances of the *Lachnospiraceae_NK4A136_group_unclassified, Roseburia, Lachnospiraceae_NK4A136_group, Clostridiales_unclassified, Roseburia_*sp_, *Lachnospiraceae*, and *Clostridiales_unclassified* belonging to phylum *Firmicutes*, as well as *Alistipes, Rikenellaceae, and Alistipes.* sp*__uncultured_Alistipes_*sp_ belonging to the phylum Bacteroidetes showed significant enrichment in the C57BL/6J-NC group (Figure 3D, also see Supplementary Figure 3B).

Functional predictions indicated that the most dominant bacterial functions were the tricarboxylic acid cycle, arginine, ornithine and proline interconversion, glycogen degradation I (cytochrome c), fatty acid &beta; -oxidation I and pyruvate fermentation in Balb/c-NC and Balb/c-5Gy groups; aerobic respiration I (cytochrome c), fatty acid &beta; -oxidation I, TCA cycle and pyruvate fermentation in Balb/c-5Gy and C57BL/6J-5Gy groups; arginine, ornithine and proline interconversion, arginine, ornithine and proline interconversion, and sucrose degradation III and TCA cycle in Balb/c-NC and C57BL/6J-NC groups; adenosine nucleotides degradation, polymyxin resistance, L-lysine biosynthesis, pyrimidine deoxyribonucleotides *de novo* biosynthesis, superpathway of tetrahydrofolate biosynthesis and salvage, urea cycle in C57BL/6J-NC and C57BL/6J-5Gy (see Supplementary Figure 4).

### Proton Irradiation Causes Alterations in Fecal Metabolite Profiles

**FIGURE 4:**
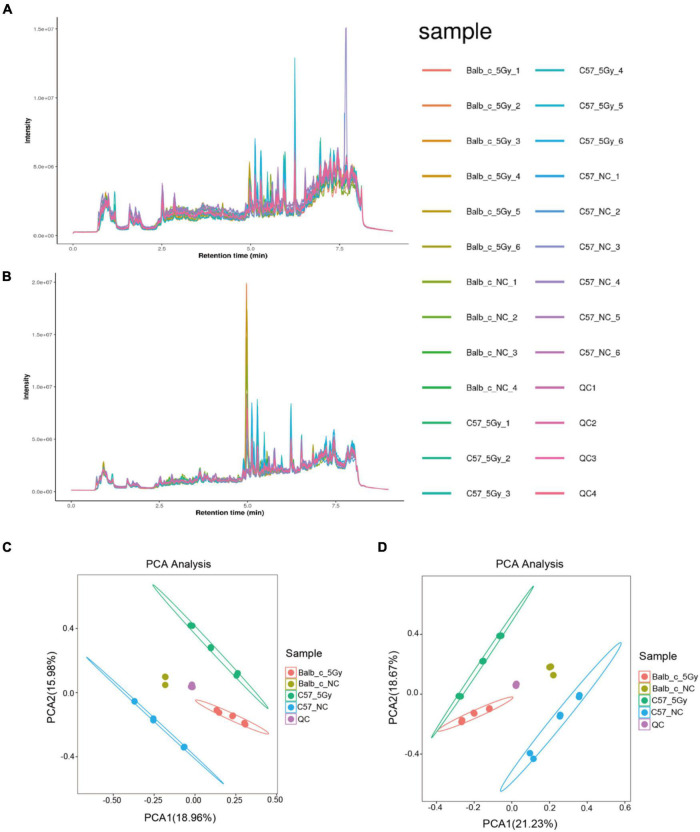
Metabolite detection quality control. Total Ion Chromatogram (TIC): overall control of the overall mass spectrum signal intensity of the sample. TIC can macroscopically reflect the separation of all metabolites in the liquid phase spectrum. **(A,C)** TIC of positive and negative. **(B,D)** PCA analysis of the identified metabolic ion. (Balb/c-NC: *n* = 3; Balb/c-5Gy: *n* = 6; C57BL/6J-NC: *n* = 6; C57BL/6J-5Gy: *n* = 6).

As suggested by 16S rRNA sequencing, the gut microbiome showed remarkable changes at 3 days post-proton irradiation. This study explored the disturbance of the fecal metabolome at 3 days post-proton irradiation by untargeted LC-MS in negative ion (ES−) as well as positive ion (ES+) mode. Figures 4A,C present diverse total ion chromatograms (TIC) in negative and positive ion modes. The TICs showed significant differences in the positive mode compared to the negative mode. However, the TICs showed no difference in the same mode between diverse groups, indicating that TICs did not directly reflect different endogenous metabolites in each group. As a result, to better discover the differences between the endogenous metabolites from the same group, this study performed PCA analysis to compare the metabolite compositions between the groups. The PCA score plots for the fecal metabolic profiles of Balb/c-NC, Balb/c-5Gy, C57BL/6J-NC, and C57BL/6J-5Gy mice are presented in negative and positive ion modes (Figures 4B,D). The dots in the PCA model represent individual fecal samples. These fecal sample groups were classified into four categories, indicating significant changes in endogenous metabolites.

**FIGURE 5:**
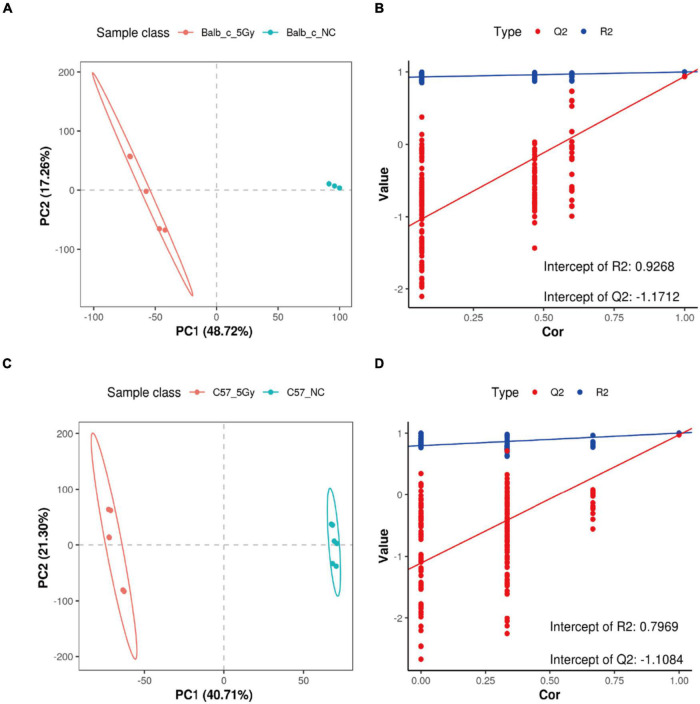
Important discriminatory metabolites identified. PLSDA analysis displaying the grouped discrimination of the Balb/c-NC, Balb/c-5Gy, C57BL/6J-NC, and C57BL/6J-5Gy groups by the first two PCs. (**A,B**: Balb/c-NC vs. Balb/c-5Gy; **C,D**: C57BL/6J-NC vs. C57BL/6J-5Gy) (Balb/c-NC: *n* = 3: Balb/c-5Gy: *n* = 6; C57BL/6J-NC: *n* = 6; C57BL/6J-5Gy: *n* = 6).

As observed from the PLS-DA score plots, the microbial profiles of Balb/c-NC, Balb/c-5Gy, C57BL/6J-NC, and C57BL/6J-5Gy groups were distinct from each other (Figures 5A–D). Two hundred permutation tests were utilized to analyze the quality of the two PLS-DA components; based on which, R2 of 0.9268 and Q2 of -1.1712 in the Balb/c-NC and Balb/c-5Gy, R2 of 0.8193 and Q2 of −0.9771 in the Balb/c-5Gy and C57BL/6J-5Gy, R2 of 0.9305 and Q2 of −1.063 in the Balb/c-NC and C57BL/6J-NC, and R2 of 0.7969 and Q2 of −1.1084 were observed in the C57BL/6J-NC and C57BL/6J-5Gy groups, respectively. Q2 represents the prediction rate of the model. For R2 and Q2, these two values are higher than 0.5, and the closer the value is to 1, the better. When the model parameters (R2 and Q2) are relatively high, the current PLS-DA model appears to be more reliable. Overall, there was a significant change in the fecal metabolome after proton irradiation. As expected, the hierarchical cluster analysis of the differential metabolites indicated that the fecal metabolome of the irradiated mice was significantly distinct from that in the non-irradiation mice (Supplementary Figure 5). Compared to the corresponding strains in the non-irradiation group, the Balb/c-5Gy group was enriched with 114 metabolites upon irradiation, while the C57BL/6J-5Gy group was enriched with 145 metabolites. Thirty-two metabolites were elevated in both of these irradiation groups, and the top 10 were 3-hydroxybenzaldehyde, PG 14:0; PG(7:0/7:0), 2-Methyl-5-(8-pentadecenyl)-1, 3-benzenediol, Piperidine, 4-hydroxybenzaldehyde, 3-Oxo-4,6-choladienoic acid, Uric acid, 17.alpha.-Dihydroequilin, Docosapentaenoic acid and beta-Zearalenol, in order of the degree of change in C57BL-6J mice (see Supplementary Tables 3–5 for details of the rest). In addition, metabolites simultaneously upregulated in both the C57BL/6J-5Gy and Balb/c groups were Ricinoleic acid, 4-Hydroxybenzaldehyde, 3-Oxooctadecanoic acid, Linoleic acid, Ortho-hydroxyphenylacetic acid.

Some of the significant differences were observed in lipids. Lipids are a component of numerous cell structures and execute various functions, including production of the cell membrane and energy storage. Recently, several articles have reported their function in inflammatory response and cell signaling (Tian et al., 2019). 5,6-Epoxy-8Z,11Z,14Z-eicosatrienoic acid represents the TRPV4 agonist, which is a strong vasodilator, calcium mobilizer, and insulin stimulator within the pancreatic islets of rats. It can resist apoptosis, fibrosis, inflammation, and cardiovascular diseases (CVDs) (Lai and Chen, 2021). It has biological actions that are essential to maintain water and electrolyte homeostasis. Imig et al. (2020) observed that vasodilation, the inhibition of the epithelial sodium channel, and the inhibition of inflammation are the major actions of 5,6-Epoxy-8Z,11Z,14Z-eicosatrienoic acid that benefit the heart, resistance arteries, and kidneys. Bougnoux et al. (2009, 2010) reported that Docosapentaenoic acid can affect the efficacy of chemotherapy and radiation therapy in patients with BC. It makes malignant tumor cells sensitive to chemotherapy and radiation therapy without increasing toxicity to non-tumor tissues (Chlebowski et al., 2006; Brasky et al., 2010). Besides, this acid increased the illustrated antihypertensive, anti-inflammatory, and organ protective actions (Imig et al., 2020). In short, of all the upregulated metabolites compounds, some of the compounds play a positive role in intestinal damage repair, especially in the scavenging function of oxygen free radicals.

### Identification of Metabolites in Each Comparison Group

**FIGURE 6:**
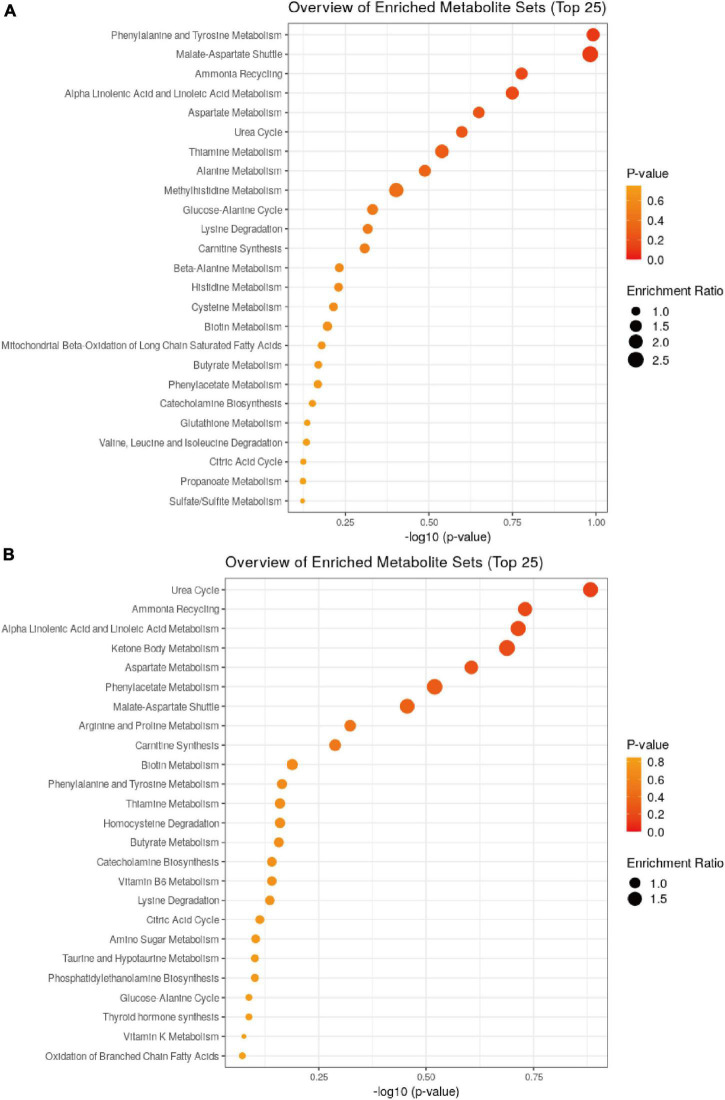
Pathway analysis for metabolites. Summary of pathway analysis for differential metabolites in ggplot2, and significantly enriched pathways are displayed by a bubble plot (*p* < 0.05, Fisher’s exact test). (**A**: Balb/c-NC vs. Balb/c-5Gy; **B**: C57BL/6J-NC vs. C57BL/6J-5Gy).

The results of the KEGG enrichment analysis using ggplot2 are displayed as a scatter plot that differed in the pairwise comparisons between the Balb/c-NC, Balb/c-5Gy, C57BL/6J-NC, and C57BL/6J-5Gy groups (Figure 6). The main pathways in C57BL/6J NC vs. 5Gy are Urea Cycle, Ammonia Recycling, Alpha Linolenic Acid and Linoleic Acid Metabolism, Ketone Body Metabolism, Aspartate Metabolism, Phenylacetate Metabolism, Malate-Aspartate Shuttle, Arginine and Proline Metabolism and Carnitine Synthesis. In the Balb/c NC vs. 5Gy group, the main pathways are Phenylalanine and Tyrosine Metabolism, Malate-Aspartate Shuttle, Ammonia Recycling, Alpha Linolenic Acid and Linoleic Acid Metabolism, Aspartate Metabolism, Urea Cycle, Thiamine Metabolism, Alanine Metabolism and Methylhistidine Metabolism. The co-enrichment pathways are Urea Cycle, Ammonia Recycling, Alpha Linolenic Acid and Linoleic Acid Metabolism, Aspartate Metabolism, and Malate-Aspartate Shuttle.

### Correlations Between the Gut Microbiome and Metabolome

**FIGURE 7:**
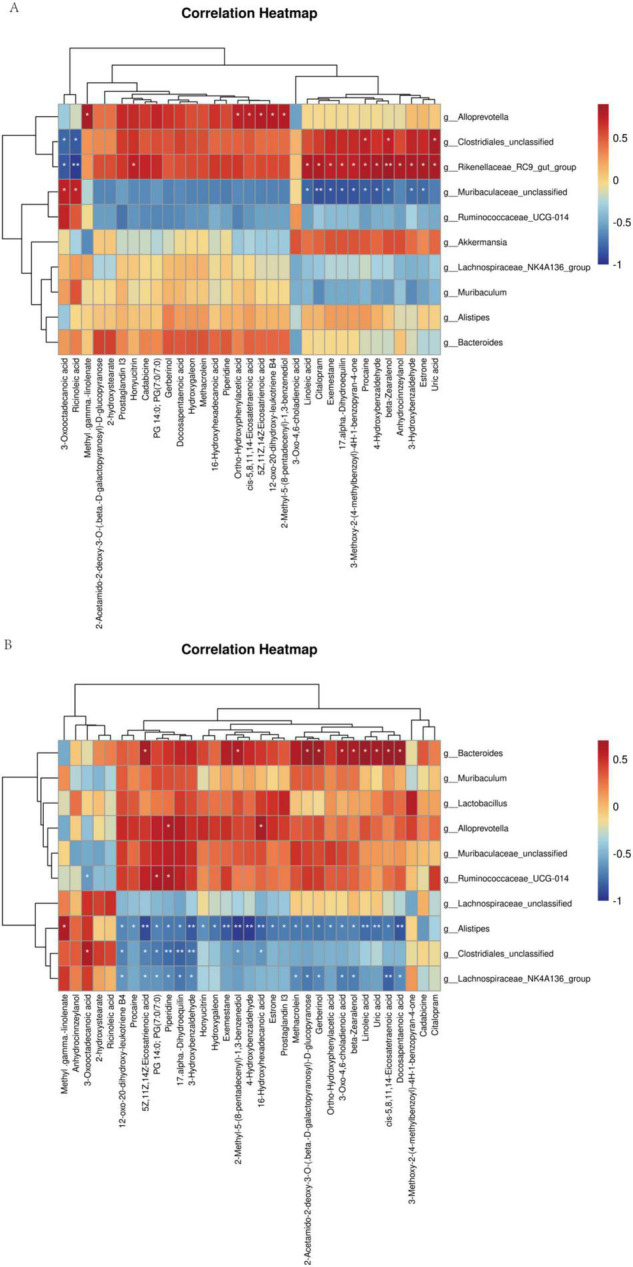
Spearman rank correlation between metabolites and bacterial genera and species. Spearman correlation between statistically different metabolites and bacterial genera was calculated both for Balb/c **(A)** and C57BL/6J **(B)**.

Spearman’s correlation analysis between significantly different metabolites and microbes in Balb/c mice and C57BL/6J mice, respectively, to obtain the relationship between metabolites and microbes (Figure 7). As a result, in balb/c mice, at the genus level, the Rikenellaceae_RC9_gut_group, Clostridiales_unclassified, and Alloprevotella, were positively correlated with most of the differential metabolites; Two groups, Muribaculaceae_unclassified, Ruminococcaceae_UCG-014, were negatively correlated with most of the differential metabolites. In C57BL/6J mice, at the genus level, Bacteroides, Muribaculaceae_unclassified, Ruminococcaceae_UCG-014, Muribaculum, Lactobacillus, and Alloprevotella were positively correlated with most of the differential metabolites. Three groups, Alistipes, Clostridiales_unclassified, and Lachnospiraceae_NK4A136_group, were negatively correlated with most of the differential metabolites. Noteworthy, comparing the above two correlation analyses, we found that the correlation between Muribaculaceae_unclassified, Ruminococcaceae_UCG-014 with metabolites was negatively correlated in Balb/c mice and positively correlated in C57BL/6J mice. Meanwhile, the correlation between Alistipes, Clostridiales_unclassified, with metabolites was positive in Balb/c mice and the opposite in C57BL/6J mice.

## Discussion

So far, there is little research that examines the impacts of proton irradiation or gut microbial manipulation, especially in mice of different species. Jiang et al. (2019) demonstrated that spaceflight significantly affected the mammalian gut microbiome. Casero et al. (2017) observed that microbial ecology was associated with signals of cell damage repair from the host in a dose-dependent manner.

In the present cross-sectional research, the NC group showed increased diversity (α-diversity) and richness of intestinal bacterial flora compared with that of the proton irradiation group. Bacteroidetes and Firmicutes are the main components of intestinal microflora. This result was in line with previous studies analyzing gut microbiota in humans and mice. As suggested by Casero et al. (2017), a high dose of linear energy transfer radiation will substantially change the gut microbial composition, as well as the α-and β-diversities.

According to the present trends, it is necessary to shift the focus from single pathogens to an ecological method wherein the community is considered as a whole to understand the effect of gut microbiota on disease and health (Coyte and Rakoff, 2019). In this regard, this work utilized high throughput 16S rDNA sequencing to examine the alterations in gut microbial composition post-irradiation. As a result, abundances of Bacteroidetes, Firmicutes, Proteobacteria, Verrucobacteria, Deferrobacterium, Cyanobacteria, Epsilonbacteraeota, and Actinobacteria, Patescibacteria, Tenericutes, Chloroflexi were changed, which was tightly associated with the radioactive intestinal damage. Li et al. (2020b) demonstrated that the probiotics Akkermansia and Lactobacillus participated in the recovery from radiation enteritis. The probiotics Lactobacillus and Akkermansia exhibited markedly increased abundances in Balb/c and C57BL/6J mice exposed to proton irradiation which implied that they were important in mitigating the intestinal damage induced by irradiation (Li et al., 2020b). In this study, the abundance of Lactobacillus was significantly reduced in Balb/c-5Gy, whereas elevated in C57BL/6J-5Gy. We hypothesize that the changes in intestinal flora upon exposure to proton irradiation vary in different strains of mice, and even gut microbes at different intervals produce different radiation responses to protect the gut.

The present work reported results based on the mouse model, aiming to delineate the gut metabolome and regulation of microbiota following proton irradiation in diverse strains of mice. Fecal microbiomes were investigated for application in mice of Balb/c-NC, Balb/c-5Gy, C57BL/6J-NC, and C57BL/6J-5Gy, and the profiles of both microbiomes were correlated. Radioactive intestinal damage was observed 3 days after proton exposure, and the abundance and structural characteristics of intestinal flora in the different mouse strains before and after exposure were visible in the samples. Different groups had diverse bacterial taxa; specifically, Muribaculaceae_unclassified was enriched in Balb/c-NC, Rikenellaceae_RC9_gut_group in Balb/c-5Gy, Lachnospiraceae_NK4A136_group in C57BL/6J-NC, and Lactobacillus in C57BL/6J-5Gy. Wang et al. (2018) found that Lactobacillus could improve colitis by enhancing intestinal barrier function and reducing inflammatory factors.

Then et al. (2020) observed that when tumors were irradiated and monitored, each mouse showed higher abundances of bacterial species belonging to the Muribaculaceae family. In this study, Muribaculaceae family bacteria unexpectedly showed decreased abundances in the Balb/c-5Gy mice compared to Balb/c-NC mice. In contrast, the increased abundance of Muribaculaceae in the C57BL/6J-5Gy group compared with C57BL/6J-NC indicated changes in the gut microbiome post-proton irradiation (Figure 3). The gut microbiome of C57BL/6J mice exposed to protons was inconsistent with studies by Jiang et al. (2019), wherein the gut microbiome, including microbial abundances, changes of community structure, and increased F/B ratios, of the RR-1 group during space flights was consistent with that of twin astronauts during their 1-year mission to the International Space Station (ISS). In the present study, there was no significant change in F/B values between the irradiated and unirradiated groups in Balb/c mice, both being 0.6, while in the C57BL/6J mice, there was a significant downward trend with 1.4 in the unirradiated group and 0.8 in the irradiated group. However, the decrease in F/B ratio was consistent with the findings of Kalkeri et al. (2021). We hypothesized a potential link between the acute intestinal injury caused by the high dose and the decrease in F/B ratio.

According to the PICRUSt function prediction analysis (Supplementary Figure 4), in Balb/c-NC, Balb/c-5Gy, C57BL/6J-NC, C57BL/6J-5Gy mice, the TCA cycle, amino acid interconversion and synthesis, DNA synthesis, and anaerobic enzyme solution pathways were widespread. In the case of high-dose LET radiation, high-dose modulatory factors can be expected, including the response of oxidative phosphorylation pathway to the microbial ecosystem and protective DNA repair (Douglas et al., 2004; Sokolov and Neumann, 2015), which are enhanced via an epithelial or local hematopoietic cell response (Burnett et al., 2013). A study by Sun et al. (2020) demonstrated that PICRUSt2 had poor predictive accuracy for mouse-related samples. Therefore, the PICRUSt function prediction can only provide us with a reference, while the signal pathway involved in the prediction needs further investigation.

Notably, the changes in Akkermansia have caught our attention. In this study, the abundance of Akkermansia in the gut of proton-irradiated mice was elevated to varying degrees, especially in Balb/c mice. As reported in the literature, BALB/c mice are susceptible to radiation and cannot tolerate the same radiation dose as C57 BL/6J mice. As reported in a previous article, the decreased abundance of Akkermansia was related to severe appendicitis. Recently, Interventions by different means lead to an increased abundance of Akkermansia, which can inhibit the development of diseases in mice or humans (Bouman and Kaur, 2021; Fan et al., 2021; Ghosh et al., 2021; Liu et al., 2021; Luo et al., 2021; Tsutsumi et al., 2021). This implies that Akkermansia may have initiated a probiotic effect. However, in the complicated intestinal ecosystem, these results may depend on the environment (Png et al., 2010; Rajilić et al., 2013). Environmental contamination represents an unavoidable problem in studies on the microbiome (Eisenhofer et al., 2019). This study did not rule out that such alterations were due to mutualistic protective responses to adverse changes.

Studies have demonstrated that gut microbiota significantly influences the flora. Although our experiment did not analyze plasma inflammatory factors, this theory has been supported by several reports. In our metabolomics analysis, it was observed that the content of Arachidonic Acid was significantly increased after proton radiation in Balb/c and C57 BL/6J mice (also see Supplementary Tables 3, 4). Arachidonic acid is a type of anti-inflammatory agent, which participates in the regulation of inflammatory, which indirectly proves our hypothesis. Wang et al. (2020) reported that during the process of regeneration after irradiation, arachidonic acid positively regulates the proliferation of intestinal epithelial cells by upregulating the expression of Ascl2 and activating WNT signaling, and simultaneously activating the radiation-resistant MSI1 cells. Huang et al. (2020) demonstrated that in irradiated HBE cells, arachidonic acid was dysregulated due to p53.

Presently, single-omics studies alone cannot sufficiently interpret the pathogenesis and progression of the disease, and it is necessary to perform comprehensive multi-omics studies.

Based on this study, several gut metabolites were changed following high linear energy proton irradiation. These findings provided indirect evidence regarding the interactions between the microbiome and the host, and more studies should be conducted to comprehensively illustrate the crosstalk between host-derived targets or signals and LDR-responsive alterations.

The changes in a range of metabolites induced by proton irradiation were also of interest to us in this study. We all know that C57BL/6J mice are radioresistant, while Balb/c mice are more sensitive to radiation. Kong et al. (2016) found that 3-hydroxybenzaldehyde inhibited inflammatory markers and signaling molecules and showed vasoprotective efficacy *in vitro* and *in vivo*. Furthermore, 3-hydroxybenzaldehyde and 4-hydroxybenzaldehyde have increased intracellular antioxidant activity, wound healing, and cell migration (Kang et al., 2017; Chen et al., 2020).

In this study, uric acid was found to be significantly elevated. Vicente et al. (2020) reported that uric acid can be involved in point-of-care biodosimetric assays after exposure, which can be used for identifying biomarkers. Pluimakers et al. (2020) reported that uric acid could be a biomarker in abdominally irradiated survivors. Paithankar et al. (2020) observed that uric acid may emerge to mitigate oxidative injury caused by irradiation in higher organisms. In additional, Paithankar et al. (2020) reported that uric acid explored potential as radio-protectors, because of survival ratio improved in human dermal fibroblast cells and may mitigate radiation-induced oxidative damage in higher organisms. Wu et al. (2020) found that the elevated uric acid directly contributed to the damage of the intestinal barrier. Afterward, we can do research around uric acid to balance the damage to the gut from radiation.

The urea cycle was found to be very forward in the metabolite enrichment pathway (Figure 6). The urea cycle is the final shared pathway for the mammalian excretion of waste nitrogen and is the primary detoxification route for the ammonia (Leonard and Morris, 2002). The urea cycle mainly involves Ornithine, citrulline and aspartic acid. Lutgens and Lambin (2007) demonstrated that plasma citrulline was a biomarker of radiation-induced epithelial damage in the small intestine. Zhao et al. (2017) demonstrated a dramatic decrease in urinary excretion of citrulline 3 days after irradiation. In conjunction with our metabolite pathway enrichment analysis, it was hypothesized that irradiation would activate the urea cycle pathway when the balance of the urea cycle was disrupted, metabolizing the excess nitrogenous material, and avoiding the development of hyperammonemia.

In the metabolite enrichment analysis, we found several amino acids with increased metabolic enrichment. Amino acids are generally considered the primary source of energy for enterocytes (Okine et al., 1995; Alpers, 2000). Various studies have shown that under normal conditions, glutamine, glutamate, and aspartate are the primary sources of energy. As shown in Figure 6, Aspartate Metabolism, Glutamate Metabolism is significantly enriched, while Phenylalanine and Tyrosine Metabolism, Lysine Degradation, Spermidine, and Spermine Biosynthesis were also significantly enriched. There is evidence that arginine supplementation to mice induces increased resistance to intestinal bacterial metastasis (Ersin et al., 2000). Exposure of the gut to radiation increases the metabolism of amino acids, so we hypothesized that some of these would contribute to the recovery of the gut, such as energy uptake and the repair of the intestinal barrier, among other effects.

Taken together, the higher or lower abundances of such bacterial species reveal that further research is necessary to illustrate the possible bacterial mechanisms in adapting to proton irradiation. Besides, with the development of manned space technology and the popularization of proton radiation therapy during the last several decades, increasing attention has been paid to proton radiation responses and the specific factor(s) resulting in such contradictory effects (adverse vs. beneficial) of proton radiation. Are the changes in the intestinal flora of mice or humans after proton radiation like other radiation sources (such as heavy ions)? What are the changes in the intestinal flora of Chinese astronauts exposed to outer space radiation? Will the changes in the intestinal flora of patients undergoing proton radiation therapy be consistent with those observed in mice? What benefit will stool transplantation treatment have on humans or mice after proton radiation? The next step in our studies will be in line with the global concern of proton irradiation to investigate the relevant medical topics.

Our work has some limitations. First, each strain of mice was classified into two groups, while irradiation was performed with only one dose, and only one time point was sampled. No comparisons were made between different doses and time points. The number of groups was small. In the future, a study design with more dosages of irradiation will be adopted, and the number of mice will be increased. Second, the present work was descriptive without providing any evidence of the basic mechanisms underlying the response to proton exposure; therefore, it was not possible to determine the exact gut microbial functions wherein the changed abundances of microbes led to compositional changes. Finally, since the effects of proton exposure on normal human tissues have been poorly studied in the past, there is a lack of relevant literature. The changes in the mouse gut microbial community observed after proton irradiation were determined by the remodeled microbial community, which may not be an accurate representation of the human intestinal microbial community, which is quite complex. Therefore, the results of this study should be interpreted with caution because they are associated with human microbiota. Despite these limitations, this study provides a concept and basic foundation to further investigate how proton radiation affects human health and provides potential targets for the prevention or prediction of radiation damage.

In summary, the LC-MS platform was used to analyze the effects of proton irradiation on metabolites in mice microflora at 3 days after exposure. Eleven gut microorganisms and 10 metabolites were significantly altered after proton exposure, while multiple signaling pathways were predicted that might be associated with the post-irradiation intestinal repair. The changes in the microbiome caused by proton exposure were not the same in different strains of mice, but some of the resulting metabolites had a positive effect on the repair of intestinal damage.

## Materials and Methods

### Animal Studies

Male C57BL/6J and BALB/c mice aged 6–8 weeks were obtained from SIPEIFU company, Beijing, China. The study protocols were approved by the Animal Ethics Committee of the Academy of Military Medicine. The mice used in this study were bred at the Animal Laboratory Division, Beijing Key Laboratory for Radiobiology, Beijing Institute of Radiation Medicine, Academy of Military Medical Sciences (AMMS), Beijing, China, according to specific guidelines. The mice were acclimatized for a week, fed with a maintained diet, and were given free access to deionized water for drinking. The animals were raised within plastic cages at 22 ± 2°C, with air humidity of 50–70%, and a 12:12 h photoperiod. Next, we randomly divided the animals into the experimental and control groups, including the control group of C57BL/6J (C57-NC), 5Gy group of C57BL/6J (C57-5Gy), the control group of BALB/c (BALB/c-NC), and the 5Gy group of BALB/c (BALB/c-5Gy). *n* = 3 for the Balb/c-NC group and *n* = 6 for the Balb/c-5Gy, C57BL/6J-NC and C57BL/6J-5Gy group. Then, we separately put the entire bodies of mice into tubes. Proton irradiation was performed at the single-particle effect experimental terminal of the 100 MeV high intensity proton cyclotron at the China Institute of Atomic Energy Research (CIAER). Mice were exposed to whole body proton irradiation for approximately 1 min each. The study duration was 3 days. The samples were collected at 3 days after irradiation. At specific periods after irradiation, we placed each mouse into a Plexiglass box that was sterilized by autoclaving to obtain fresh fecal samples. Under aseptic conditions, the feces was transferred to a sterile ep tube (AXYGEN, Corning), aliquoted, and placed quickly into liquid nitrogen for quick freezing, and then stored at −80°C for transportation using dry ice. The pellets were shipped to the LC-Biotechnology Co., Ltd. (Hangzhou, Zhejiang Province, China) for the sequencing of the 16S rRNA amplicons.

### Isolation of Fecal DNA for Microbiome Analysis

We utilized the E.Z.N.A. Stool DNA Kit (D4015, Omega, Inc., United States) to extract fecal genomic DNA according to the specific protocols. Notably, the reagent used for extracting DNA from the sample was effective in preparing DNA from several bacterial strains. Thereafter, we eluted the collected total DNA into 50 μL of the elution buffer provided in the same kit, followed by preservation at −80°C before subjecting the samples to PCR at the LC-Biotechnology Co., Ltd., Hangzhou, Zhejiang Province, China.

### Sequencing of the Bacterial 16S rRNA

We assessed the quality of the extracted DNA by agarose gel electrophoresis (AGE) and quantified the DNA using a UV spectrophotometer. Then, barcode-indexed primers were utilized to amplify the bacterial 16S rRNA gene between its V3–V4 hypervariable regions, including 806R (5′-GGACTACHVGGGTWTCTAAT-3′) and 338F (5′-ACTCCTACGGGAGGCAGCAG-3′). Then, we used AMPure XT beads (Beckman Coulter Genomics, Danvers, MA, United States) to purify the PCR products and used Qubit (Invitrogen, United States) for product quantification. We also produced the amplicon pools to perform the sequencing analysis. Later, we evaluated the quality of the amplicon library using the Library Quantification Kit for Illumina (Kapa Biosciences, Woburn, MA, United States) and assessed its size using an Agilent 2100 Bioanalyzer (Agilent, United States). Finally, the NovaSeq PE250 platform was utilized for library sequencing.

### Determination of Fecal Metabolomics

After collection, each sample was thawed on ice, and the metabolites were isolated using 50% methanol solution. Later, the extraction mixture was stored at −20°C overnight. Then, the samples were centrifuged for 20 min at 4,000 *g* and stored at −80°C before analysis by LC-MS. We combined each extraction mixed solution (10 μL) to prepare the pooled quality control (QC) sample.

### LC-MS Analysis

Each sample was analyzed using the TripleTOF 5600 Plus high-resolution tandem mass spectrometer (SCIEX, Warrington, United Kingdom) in both negative and positive ion modes. We obtained the MS data in the mode of IDA. During the entire process of acquisition, we calibrated the mass accuracy based on 20 samples.

### 16S rRNA Statistical Analysis

The Illumina NovaSeq platform (LC-Bio) was used for sample sequencing according to the specific protocols. The paired-end reads were assigned for samples according to the specific barcode, after which primer sequences were cut off and barcoded to truncate them. Then, we adopted FLASH to merge the paired-end reads. Raw reads were subjected to quality filtering under certain conditions to obtain high-quality clean tags in line with fqtrim (v0.94). The software Vsearch (v2.3.4) was used to filter chimeric sequences. Later, DADA2 was utilized for de-replication, and a feature sequence with the feature table was obtained. Then, the reads were normalized to identical sequences at random to calculate their α and β-diversities. Later, based on the SILVA (release 132) classifier, we normalized the feature enrichment based on the relative sample abundance. The program QIIME2 was used to calculate the α-diversity indices that evaluated the community diversity (the Simpson and Shannon indices) and the richness of gut microbial communities (observed species and Chao1 indices), whereas the statistical package R was used to draw graphs. The sequences were aligned using the alignment tool BLAST; for every typical sequence, the feature sequences were annotated based on the SILVA database. Moreover, the R package (v3.5.2) was also used for creating other diagrams. We also analyzed Principal Component Analysis (PCA) based on Bray-Curtis distance to compare the global microbial compositions pre-and post-intervention for the individual groups at the operational taxonomic unit (OTU), genus, and phylum levels. Besides, the Wilcoxon rank-sum test was adopted to analyze the sample data for the above-mentioned indices, screen the distinct heterogeneities in α-diversity indices from comparisons of diverse groups, and draw the violin chart.

### Metabolics Statistical Analysis

For the processing of metabolomics data, the software XCMS was used to process the obtained LC-MS data. The ions were detected, respectively, through integrative data regarding m/z and retention time. Thereafter, such data were matched to the public and in-house databases. We eliminated features examined from <80% of the test samples or <50% of the QC samples and adopted the k-nearest neighbor algorithm to extrapolate the missing peak values to improve the data quality. We normalized the group datasets before analysis. Data on each sample were normalized by adopting the probabilistic quotient normalization algorithm.

The global distributions of the microbial metabolites of individual groups were analyzed by Partial Least Squares Discriminant Analysis (PLS-DA) in combination with unit variance scaling. Q2 (indicating model predictability) and R2X (the overall variations were interpreted by the model) were utilized to assess the quality of the PLS-DA model. Moreover, the cross-validation analysis of variance (CV-ANOVA) was conducted to validate the significance of the model. This study also carried out the univariate analysis and adjusted *p*-values for multiple testing, using the Benjamin–Hochberg method.

To conduct PERMANOVA and PCA, the ‘vegan’ and ‘ape’ functions in the statistical package R were used.

## Data Availability Statement

The data presented in the study are deposited in the NCBI repository, accession number PRJNA794301.

## Ethics Statement

The animal study was reviewed and approved by Academy of Military Medical Sciences.

## Author Contributions

YCL conceived the study, analyzed the data, and wrote the manuscript. LS contributed resources and analyzed the data. HLZ, WZ, WXH, XCL, and MS prepared figures and collected samples. YHG, FQK, and QJW performed proton irradiation experiments. HG and PKZ conceived the study and revised the manuscript critically for important intellectual content. All authors edited the manuscript and approved the final draft.

## Conflict of Interest

The authors declare that the research was conducted in the absence of any commercial or financial relationships that could be construed as a potential conflict of interest.

## Publisher’s Note

All claims expressed in this article are solely those of the authors and do not necessarily represent those of their affiliated organizations, or those of the publisher, the editors and the reviewers. Any product that may be evaluated in this article, or claim that may be made by its manufacturer, is not guaranteed or endorsed by the publisher.
