# Supplementary material for: Differences in the Establishment of Gut Microbiota and Metabolome Characteristics Between Balb/c and C57BL/6J Mice After Proton Irradiation
Source: Front Microbiol. 2022 May 6;13:874702. doi: 10.3389/fmicb.2022.874702 (PMC9157390; doi:10.3389/fmicb.2022.874702)
Supplement: Supplementary file 1 [file Data_Sheet_1.docx]

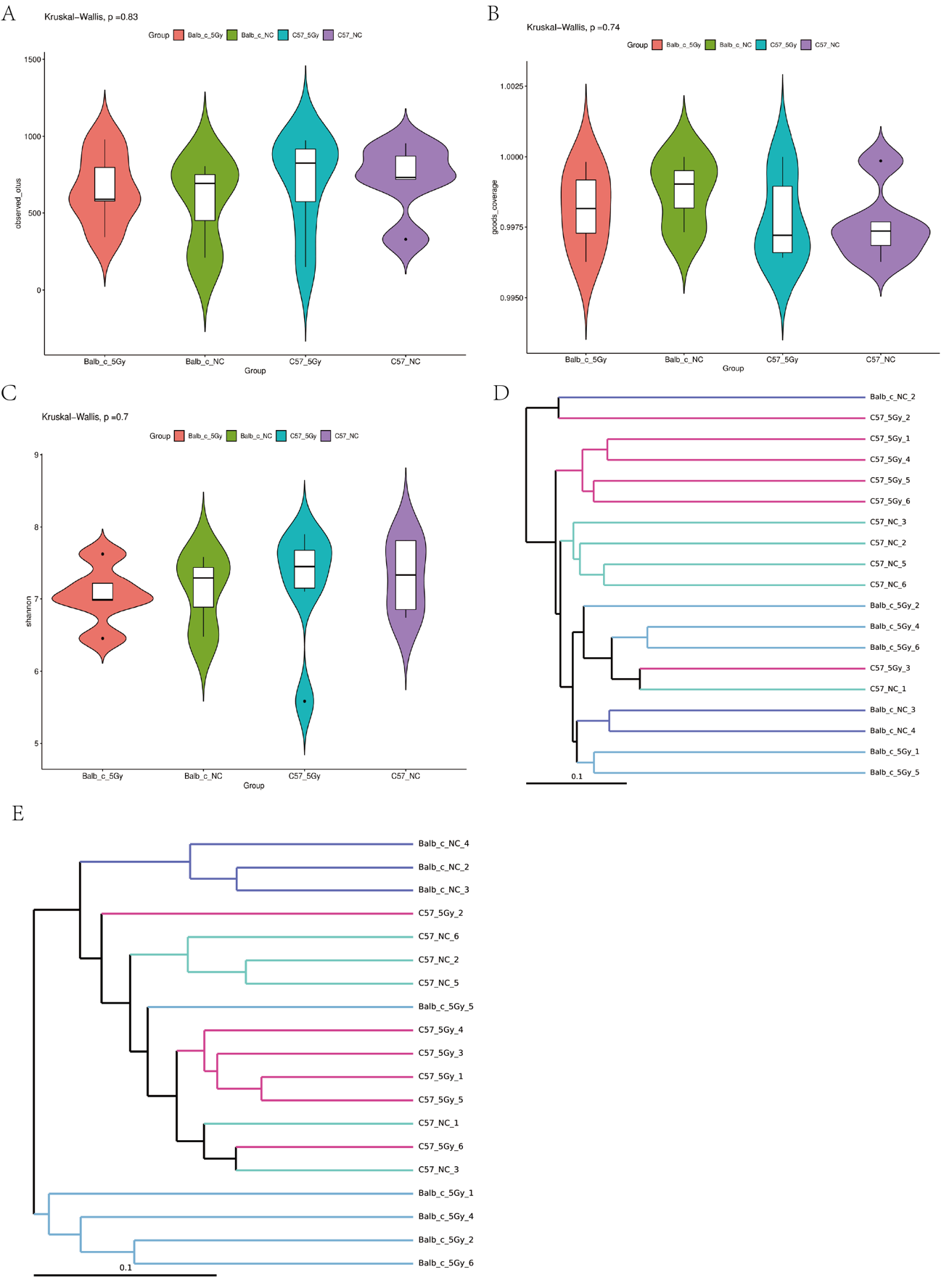
Supplement Figure 1

**Changes of gut microbiota after proton irradiation in each group at the phylum level.** The number of Observed OTU (A), Alpha diversity assessed by (B) richness (goods_coverage), and (C) evenness (Shannon) diversity of the intestinal bacteria in

male mice at days 3 post-proton irradiation were examined by 16S high-throughput sequencing. (Wilcoxon, *: p<0.05). Hierarchical cluster based on unweighted(D) and weighted(E) UniFrac distance of gut microbiota in irradiated and unirradiated groups displayed at the phylum level.


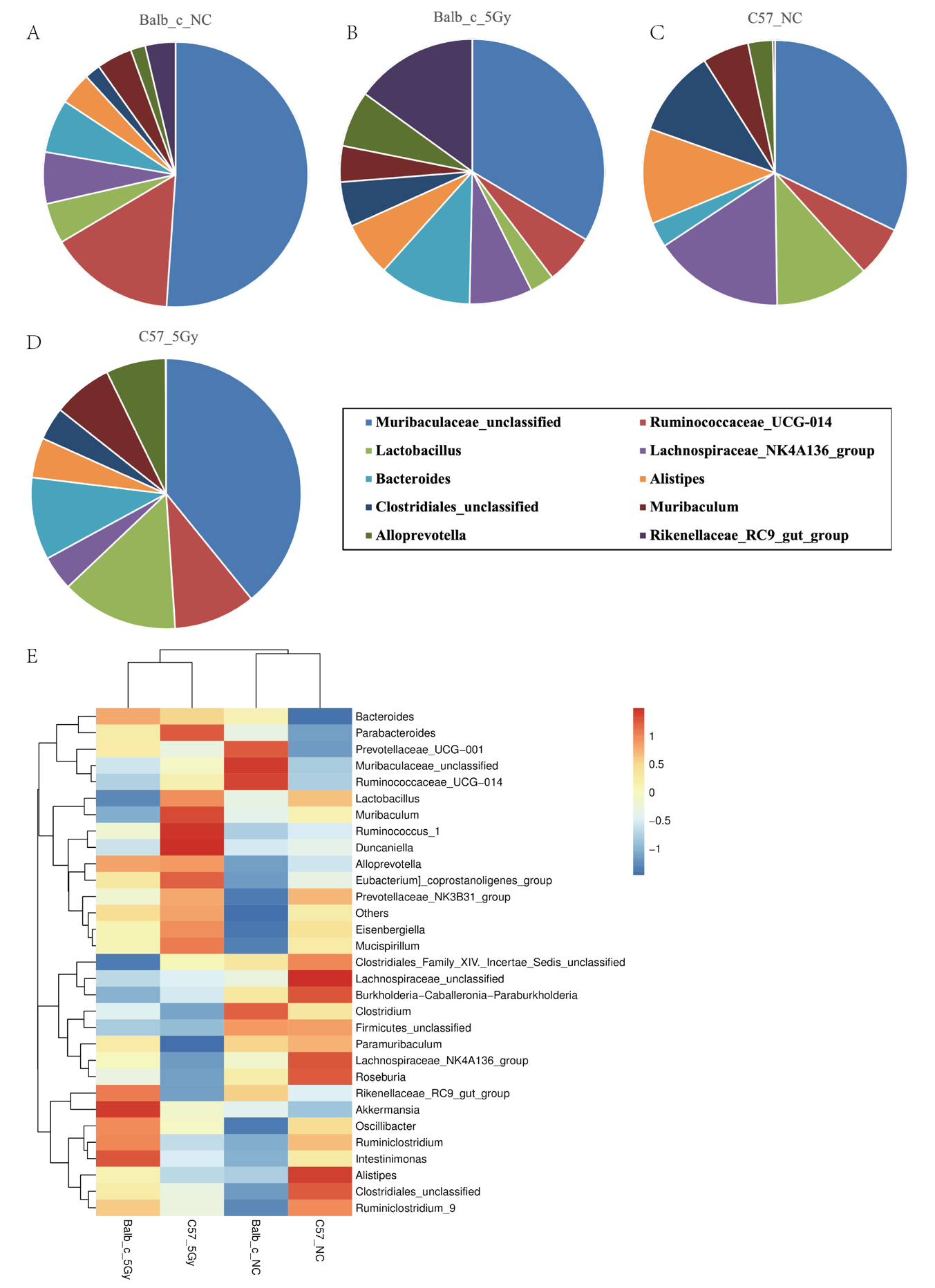
Supplement Figure 2

**Changes of gut microbiota after proton irradiation in each group at the genus level.** Average abundance of bacterial phyla in the Balb/c-NC (A), Balb/c-5Gy (B), C57BL/6J-NC (C), and C57BL/6J-5Gy (D) mice intestinal microbiota. (E) Heatmap analysis of gut microbiota changes from different mice group at genus level. (Balb/c-NC: n=3; Balb/c-5Gy: n=6; C57BL/6J-NC: n=6; C57BL/6J-5Gy: n=6.)


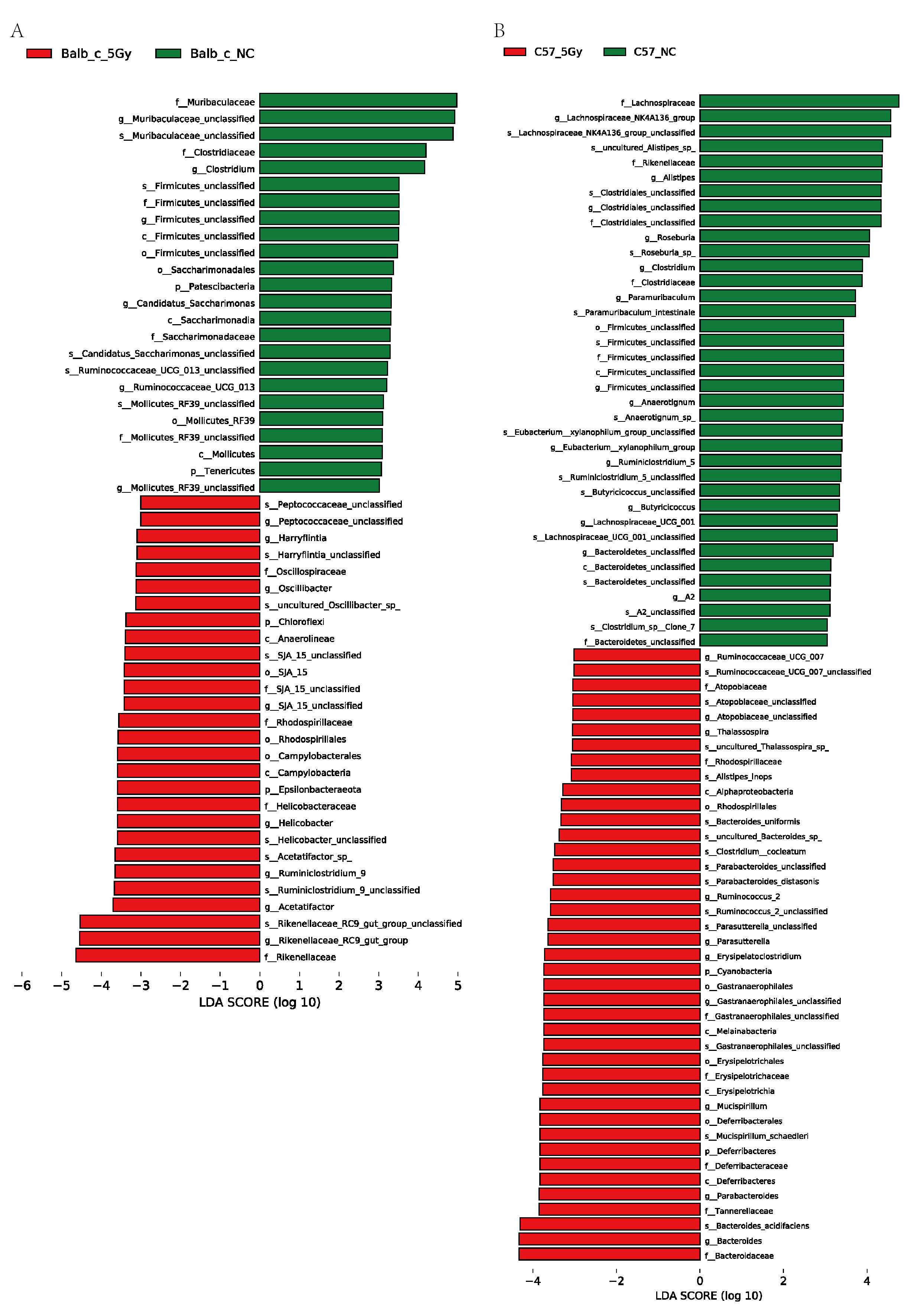
Supplement Figure3

**Cladogram and liner discriminant analysis (LDA) by LEfSe analysis showing the biomarker taxa associated with Balb/c and C57BL/6J group.** Dot size is proportional to the abundance of the taxon. (A)Green indicates taxa enriched in the Balb/c-NC group, while red indicates the taxa enriched in the Balb/c-5Gy group. (B) Green indicates taxa enriched in the C57BL/6J -5Gy group, while red indicates the taxa enriched in the Balb/c-5Gy group.


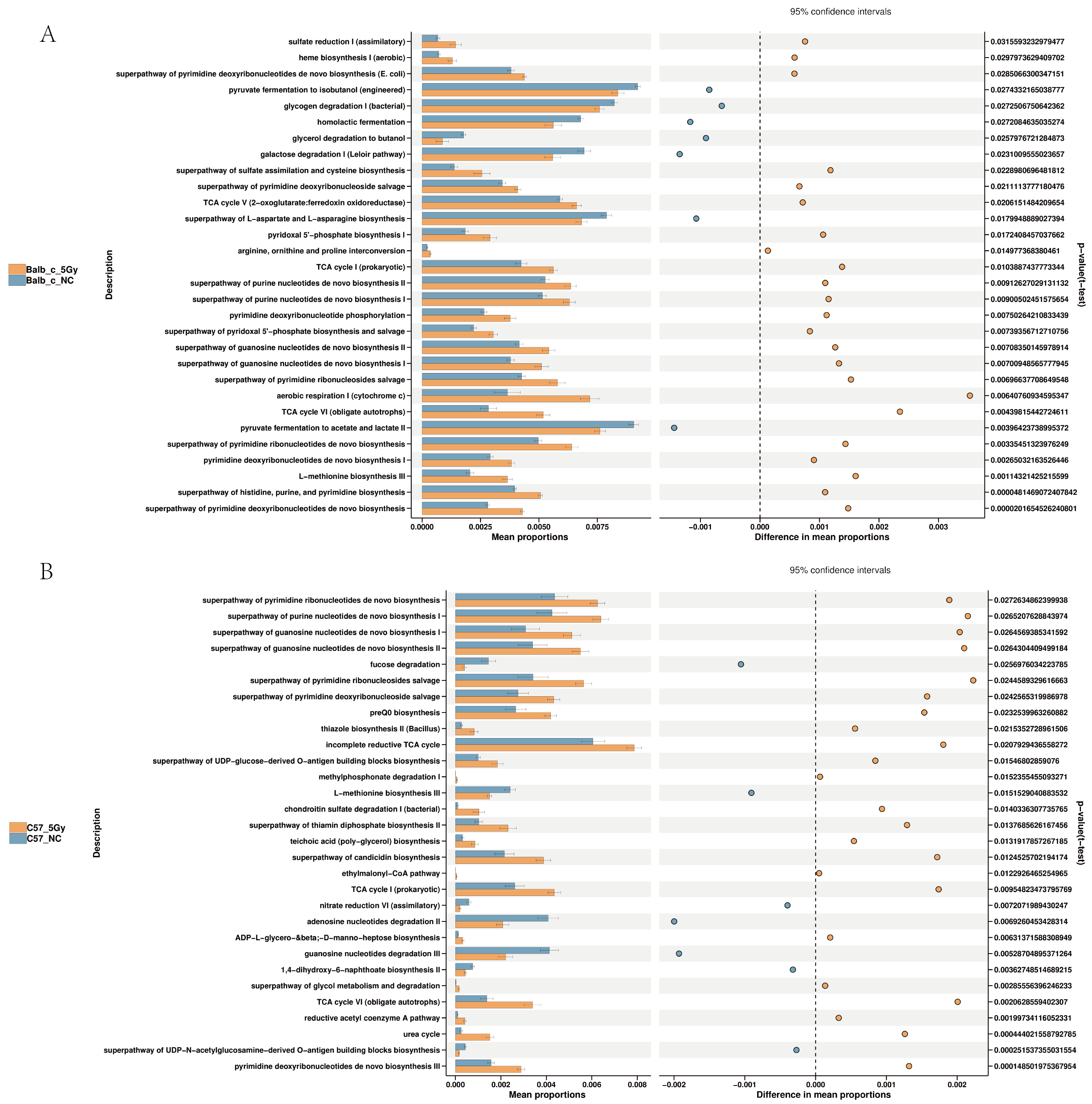
Supplement Figure 4

Functionally predicted dominant bacterial differing in proportions in groups of (A) Balb/c-NC Vs. Balb/c-5Gy, (B) Balb/c-5Gy Vs. C57BL/6J in pathway. The bar plot shows mean proportions of differential bacterial function pathways predicted using PICRUSt2. The difference in proportions between the groups is shown with 95% confidence intervals. Only p value < 0.05 (T test).

Supplement Figure 5


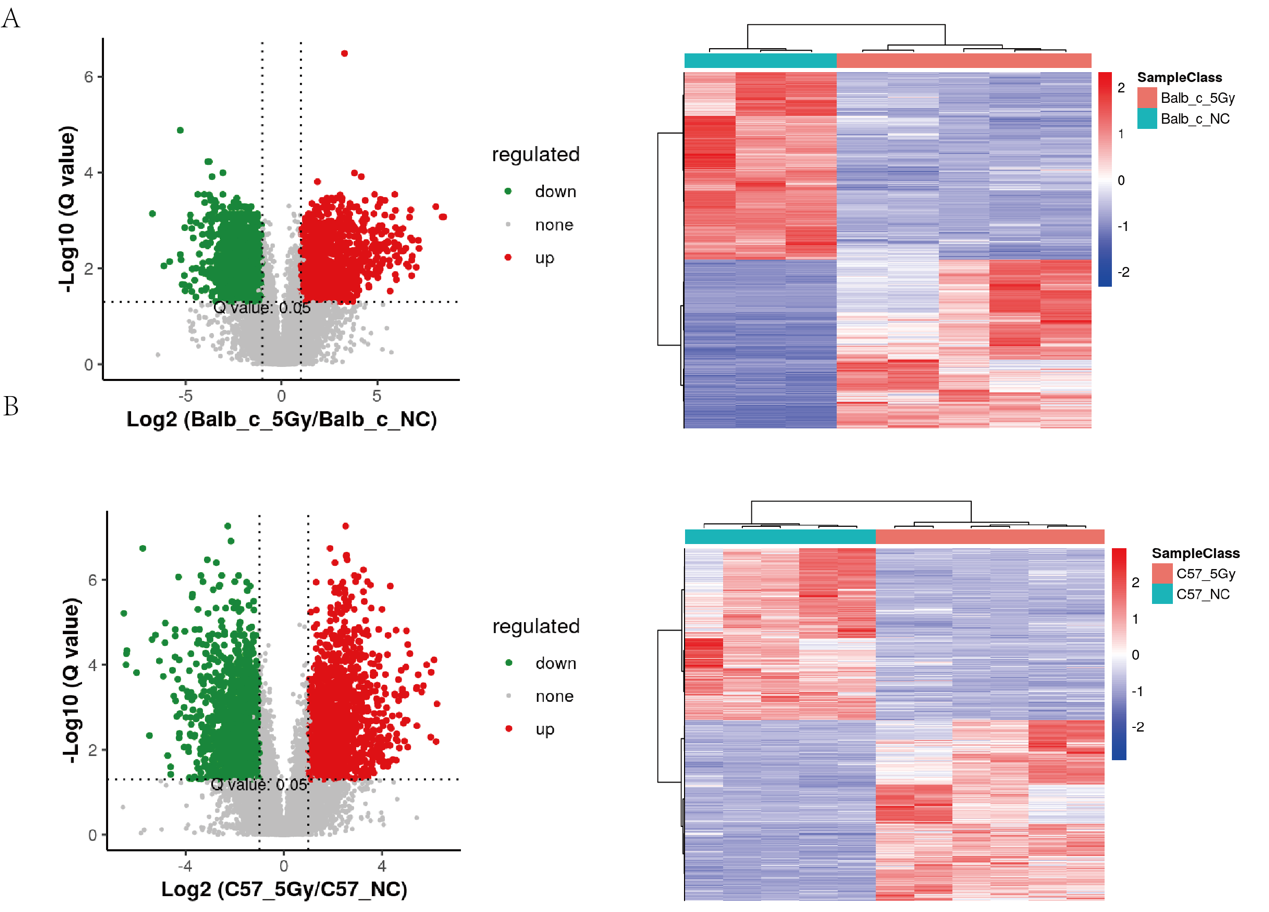


**Volcano plot and heatmap of the significant metabolites compared to group differences.** The similarities and differences between all samples are reflected in the separation and aggregation trends of the samples in the score graph. (A: Balb/c-NC vs Balb/c-5Gy; B: C57BL/6J-NC vs C57BL/6J-5Gy)


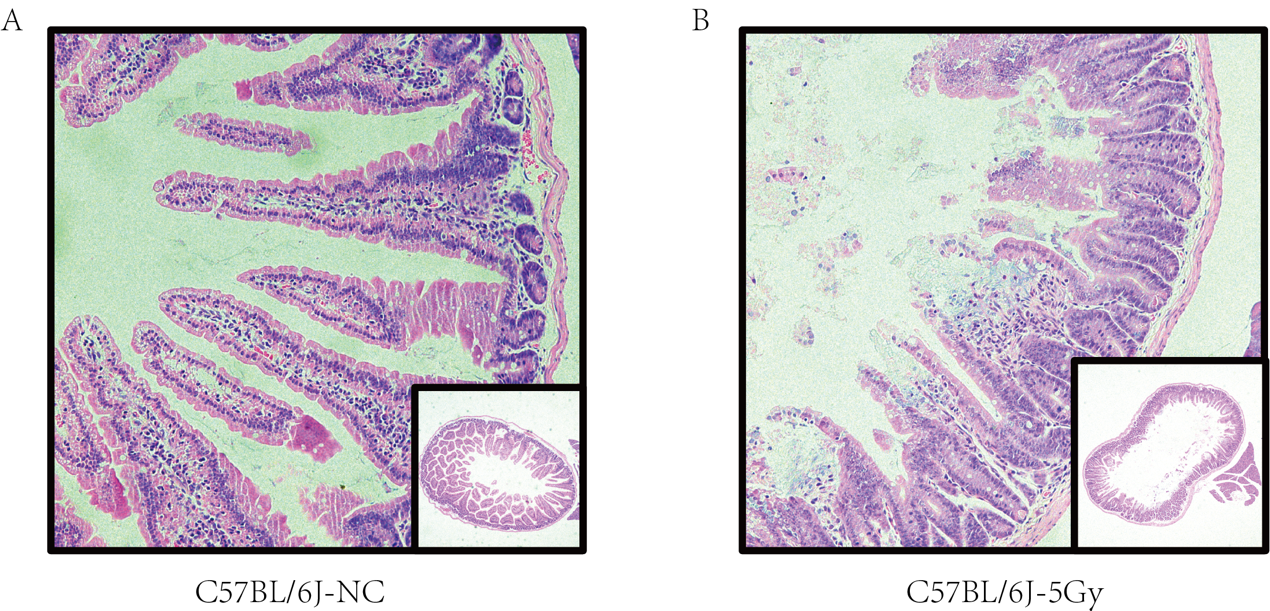
Supplement Figure 6

**Proton radiation-induced histological alterations during intestinal injury.** H&E staining of intestine 3 days after non-irradiation(A) or 5Gy(B) of total-body irradiation (TBI) in C57BL/6J mice.

Supplement table 1

Detailed list of microbiotas with changes in relative abundance for Balb/c-NC, Balb/c-5Gy, C57BL/6J-NC and C57BL/6J-5Gy in phylum.

| Legend | Taxonomy | Total | | Balb/c-NC | Balb/c-5Gy | C57BL/6J-NC | C57BL/6J-5Gy |
| --- | --- | --- | --- | --- | --- | --- | --- |
|  |  | cout | % | % | % | % | % |
|  | Bacteroidetes | 481920 | 50.21 | 59.35 | 55.75 | 40.76 | 51.79 |
|  | Firmicutes | 419940 | 43.75 | 38.24 | 34.63 | 55.40 | 42.49 |
|  | Proteobacteria | 21326 | 2.22 | 1.22 | 2.37 | 2.51 | 2.31 |
|  | Verrucomicrobia | 13888 | 1.45 | 0.03 | 4.49 | 0.01 | 0.06 |
|  | Deferribacteres | 8226 | 0.86 | 0.08 | 0.49 | 0.59 | 1.71 |
|  | Cyanobacteria | 5153 | 0.54 | 0.12 | 0.61 | 0.13 | 1.04 |
|  | unclassified | 4020 | 0.42 | 0.37 | 0.57 | 0.39 | 0.33 |
|  | Epsilonbacteraeota | 2168 | 0.23 | 0.00 | 0.71 | 0.00 | 0.02 |
|  | Actinobacteria | 1659 | 0.17 | 0.12 | 0.29 | 0.09 | 0.12 |
|  | Patescibacteria | 775 | 0.08 | 0.33 | 0.04 | 0.03 | 0.07 |
|  | Tenericutes | 527 | 0.05 | 0.13 | 0.01 | 0.08 | 0.04 |
|  | Chloroflexi | 139 | 0.01 | 0.01 | 0.04 | 0.00 | 0.00 |
|  | Acidobacteria | 112 | 0.01 | 0.00 | 0.00 | 0.02 | 0.02 |

Supplement table 2

Detailed lists of microbiotas with changes in abundance for Balb/c-5Gy vs Balb/c-NC and C57BL/6J-5Gy vs C57BL/6J-NC using the Mann-Whitney U test in phylum and genus level, sorted by p-value. FC rounded to 2 digits after the decimal point. FC: fold change.

| Balb/c-5Gy vs Balb/c-NC | | | | |
| --- | --- | --- | --- | --- |
| Genus | log2FC | wilcox.test.p_value | significance | regulation |
| g__Ruminococcaceae_UCG-013 | -6.74 | 0.02 | yes | down |
| g__Mollicutes_RF39_unclassified | -4.13 | 0.02 | yes | down |
| g__Harryflintia | Inf | 0.02 | yes | up |
| g__Anaerovorax | Inf | 0.02 | yes | up |
| g__Muribaculaceae_unclassified | -0.81 | 0.03 | yes | down |
| g__Rikenellaceae_RC9_gut_group | 1.81 | 0.03 | yes | up |
| g__Clostridium | -1.76 | 0.03 | yes | down |
| g__Ruminiclostridium_9 | 1.58 | 0.03 | yes | up |
| g__Firmicutes_unclassified | -1.10 | 0.03 | yes | down |
| g__Acetatifactor | 4.01 | 0.03 | yes | up |
| g__Peptococcaceae_unclassified | 2.16 | 0.03 | yes | up |
| g__Helicobacter | 7.37 | 0.03 | yes | up |
| g__Candidatus_Saccharimonas | -3.21 | 0.03 | yes | down |
| g__SJA-15_unclassified | 1.43 | 0.03 | yes | up |

| C57BL/6J-5Gy vs C57BL/6J-NC | | | | |
| --- | --- | --- | --- | --- |
| Phylum | log2FC | wilcox.test.p_value | significance | regulation |
| p__Cyanobacteria | 3.02 | 0.02 | yes | up |
| p__Verrucomicrobia | 2.95 | 0.03 | yes | up |
| p__Epsilonbacteraeota | Inf | 0.03 | yes | up |
| p__Deferribacteres | 1.53 | 0.04 | yes | up |
| p__Firmicutes | -0.38 | 0.10 | no | down |
| p__Bacteroidetes | 0.35 | 0.20 | no | up |
| p__Actinobacteria | 0.46 | 0.27 | no | up |
| p__Patescibacteria | 1.40 | 0.27 | no | up |
| p__Planctomycetes | -Inf | 0.27 | no | down |
| p__Acetothermia | -Inf | 0.27 | no | down |
| p__Armatimonadetes | -Inf | 0.27 | no | down |
| p__Candidatus_Aminicenantes | -Inf | 0.27 | no | down |
| p__Tenericutes | -1.11 | 0.36 | no | down |
| p__Nitrospirae | 1.20 | 0.40 | no | up |
| p__Chloroflexi | -1.32 | 0.82 | no | down |
| p__Proteobacteria | -0.12 | 0.86 | no | down |
| p__Acidobacteria | 0.05 | 0.86 | no | up |
| p__unclassified | -0.23 | 1 | no | down |

| C57BL/6J-5Gy vs C57BL/6J-NC | | | | |
| --- | --- | --- | --- | --- |
| Genus | log2FC | wilcox.test.p_value | significance | regulation |
| g__Paramuribaculum | -3.85 | 0.01 | yes | down |
| g__Parabacteroides | 4.32 | 0.01 | yes | up |
| g__Parasutterella | 4.98 | 0.01 | yes | up |
| g__Alistipes | -1.26 | 0.01 | yes | down |
| g__Erysipelatoclostridium | 3.47 | 0.01 | yes | up |
| g__Ruminiclostridium_5 | -2.20 | 0.01 | yes | down |
| g__Lachnospiraceae_UCG-001 | -3.74 | 0.01 | yes | down |
| g__UBA1819 | -2.35 | 0.01 | yes | down |
| g__Peptococcus | -3.75 | 0.01 | yes | down |
| g__Thalassospira | 4.29 | 0.01 | yes | up |
| g__Christensenellaceae_R-7_group | 1.89 | 0.01 | yes | up |
| g__Elizabethkingia | -Inf | 0.01 | yes | down |
| g__Rhodospirillales_unclassified | Inf | 0.01 | yes | up |
| g__Ruminococcaceae_UCG-008 | Inf | 0.01 | yes | up |
| g__Atopobiaceae_unclassified | Inf | 0.01 | yes | up |
| g__Ruminococcaceae_UCG-010 | -2.83 | 0.02 | yes | down |
| g__Sphingopyxis | -3.27 | 0.02 | yes | down |
| g__Dehalobacterium | -2.47 | 0.02 | yes | down |
| g__Gastranaerophilales_unclassified | 3.02 | 0.02 | yes | up |
| g__Christensenellaceae_unclassified | 1.47 | 0.02 | yes | up |
| g__Family_XIII_AD3011_group | 1.35 | 0.02 | yes | up |
| g__DTU014_unclassified | 5.87 | 0.02 | yes | up |
| g__Akkermansia | 2.95 | 0.03 | yes | up |
| g__Butyricicoccus | -2.78 | 0.03 | yes | down |
| g__Lachnospiraceae_NK4A136_group | -1.93 | 0.03 | yes | down |
| g__Roseburia | -4.72 | 0.03 | yes | down |
| g__Anaerotignum | -1.59 | 0.03 | yes | down |
| g__Eubacterium]_xylanophilum_group | -1.27 | 0.03 | yes | down |
| g__A2 | -2.12 | 0.03 | yes | down |
| g__Eubacterium]_nodatum_group | 2.39 | 0.03 | yes | up |
| g__Family_XIII_UCG-001 | -1.47 | 0.03 | yes | down |
| g__Ruminococcus_2 | Inf | 0.03 | yes | up |
| g__Helicobacter | Inf | 0.03 | yes | up |
| g__Ruminococcaceae_UCG-007 | Inf | 0.03 | yes | up |
| g__Bacteroidetes_unclassified | -Inf | 0.04 | yes | down |
| g__Tyzzerella_3 | -3.76 | 0.04 | yes | down |
| g__Oscillospira | -Inf | 0.04 | yes | down |
| g__Bacteroides | 1.70 | 0.04 | yes | up |
| g__Clostridiales_unclassified | -1.42 | 0.04 | yes | down |
| g__Clostridium | -1.51 | 0.04 | yes | down |
| g__Mucispirillum | 1.53 | 0.04 | yes | up |
| g__Firmicutes_unclassified | -1.14 | 0.04 | yes | down |
| g__Lachnospiraceae_UCG-006 | -1.03 | 0.04 | yes | down |
| g__Anaerovorax | 2.34 | 0.05 | yes | up |

Supplement table 3

The details list of significant upregulated Metabolites in Balb/c-5Gy compared with Balb/c-NC, ordered depending on FC. FC rounded 2 numbers after the decimal point. FC: Fold change.

| Metabolite | FC | t.test_p.value_BHcorrect | regulated |
| --- | --- | --- | --- |
| Mesobilirubinogen | 42.1809497 | 6.35E-07 | up |
| N-Oleoyl tyrosine | 28.2017606 | 3.90E-06 | up |
| Leu-Val | 25.9642117 | 0.00014042 | up |
| N-Palmitoyl tyrosine | 25.3494567 | 2.99E-05 | up |
| Piperidine | 23.1548802 | 0.00290607 | up |
| 3-Hydroxy-4-methoxycinnamic acid | 20.7833916 | 0.00033091 | up |
| Allose | 15.2556214 | 0.00599201 | up |
| N-Acetylasparagine | 15.1216157 | 0.00127399 | up |
| Prostaglandin I3 | 15.0507021 | 0.00016285 | up |
| trans-Ferulic acid | 15.0290518 | 0.00380186 | up |
| 2-Hydroxyhippuric acid | 11.8634615 | 9.86E-07 | up |
| Leucyl-Leucine | 10.1718736 | 1.66E-05 | up |
| 3-Hydroxybenzaldehyde | 10.1059635 | 0.00030442 | up |
| 1H-Pyrrole-2-carboxaldehyde | 9.85502203 | 0.00452044 | up |
| 6-Hydroxynicotinic acid | 9.76003227 | 0.00215989 | up |
| 12-oxo-20-dihydroxy-leukotriene B4 | 9.35703376 | 5.11E-05 | up |
| Prostaglandin I3 | 8.62640279 | 1.26E-06 | up |
| Sedoheptulosan | 8.26376272 | 4.04E-05 | up |
| 2-Aminoisobutyric acid | 8.05411834 | 0.00023155 | up |
| C17_Sphingosine | 7.97994776 | 0.0039365 | up |
| 4-Imidazoleacrylic acid | 7.95186443 | 0.00045143 | up |
| 17.alpha.-Dihydroequilin | 7.59396336 | 0.0006209 | up |
| Tyramine | 7.45519665 | 0.00961752 | up |
| 5-Keto-D-gluconic acid | 6.93921354 | 0.00055628 | up |
| trans-Caffeic acid | 6.8685285 | 0.0038488 | up |
| LysoPE 15:0 | 6.83386869 | 0.00363455 | up |
| LysoPE 15:0 | 6.66175469 | 0.00281733 | up |
| Allose | 6.2197586 | 2.09E-06 | up |
| 2-Hydroxy-2-(2-oxopropyl)butanedioic acid | 6.18912545 | 0.00047967 | up |
| 4-Methyl-5-thiazoleethanol | 6.12287956 | 0.00023803 | up |
| Erlose | 5.7034241 | 0.00022453 | up |
| Val-Val | 5.62914183 | 0.0006843 | up |
| beta-Zearalenol | 5.51854792 | 0.00336454 | up |
| Thiamine | 5.51773403 | 0.0008042 | up |
| 4-Hydroxystyrene | 5.45407578 | 0.00021177 | up |
| N,N'-Diacetylchitobiose | 5.40138957 | 3.84E-06 | up |
| 4-Bromo-2,6-di-tert-butylphenol | 5.24494909 | 0.00911344 | up |
| Ricinoleic acid | 5.22499302 | 4.09E-06 | up |
| 5-Hexyltetrahydro-2-furanoctanoic acid | 5.16095361 | 4.24E-07 | up |
| 4-Methyl-5-thiazoleethanol | 5.15760567 | 0.0084734 | up |
| DErySphingosine | 4.97733567 | 0.00660046 | up |
| 4-Hydroxybenzaldehyde | 4.95024142 | 0.00019518 | up |
| Palmitoyl ethanolamide | 4.89418561 | 0.001663 | up |
| Estrone | 4.87415133 | 0.000226 | up |
| (-)-N-Acetylneuraminic acid | 4.82459851 | 0.00164317 | up |
| Methacrolein | 4.82419314 | 0.00298812 | up |
| Linoleic acid | 4.75149752 | 1.06E-06 | up |
| LysoPE 16:0 | 4.70818098 | 0.00632426 | up |
| Val-Phe | 4.70250484 | 0.00254359 | up |
| Urocanic acid | 4.64168828 | 0.00427463 | up |
| PG 14:0; PG(7:0/7:0) | 4.63477761 | 0.00285456 | up |
| Exemestane | 4.59226456 | 0.0016186 | up |
| Uric acid | 4.58190247 | 0.00315172 | up |
| Honyucitrin | 4.5033007 | 0.00011306 | up |
| 4-Methyl-5-thiazoleethanol | 4.46740543 | 0.00018746 | up |
| Anhydrocinnzeylanol | 4.42355885 | 0.00406583 | up |
| Cadabicine | 4.41683743 | 0.00080497 | up |
| Erlose | 4.33658042 | 0.00451327 | up |
| Ricinoleic acid | 4.23771672 | 5.89E-06 | up |
| LysoPE 16:0 | 4.18189032 | 0.00697662 | up |
| 2-Methyl-5-(8-pentadecenyl)-1,3-benzenediol | 4.14596712 | 0.00572802 | up |
| Urocanic acid | 4.12233537 | 0.00116104 | up |
| L-Proline | 4.11662889 | 0.00974505 | up |
| 5,6-Epoxy-8Z,11Z,14Z-eicosatrienoic acid | 4.0378132 | 0.00014036 | up |
| Hydroxygaleon | 3.90525986 | 7.28E-05 | up |
| 3-Hydroxypicolinic acid | 3.87084364 | 0.00025479 | up |
| 2'-Hydroxy-2,3,5'-trimethoxychalcone | 3.84155196 | 0.00983529 | up |
| 16-Hydroxyhexadecanoic acid | 3.78794026 | 0.00731155 | up |
| 3-Oxooctadecanoic acid | 3.75435929 | 0.00458021 | up |
| Ortho-Hydroxyphenylacetic acid | 3.7449754 | 8.60E-05 | up |
| 2-hydroxystearate | 3.71859058 | 2.03E-05 | up |
| Lysine | 3.71366413 | 0.0029716 | up |
| 3-Oxo-4,6-choladienoic acid | 3.63914365 | 0.00271518 | up |
| 3-Hydroxybutyric acid | 3.5820238 | 0.00030312 | up |
| Uric acid | 3.51963579 | 0.00543603 | up |
| Prostaglandin I3 | 3.50167239 | 2.55E-05 | up |
| D-Pipecolinic acid | 3.49759275 | 0.00403331 | up |
| Uric acid | 3.45489131 | 0.0033436 | up |
| Docosapentaenoic acid | 3.40679902 | 0.00424759 | up |
| Gerberinol | 3.35226821 | 0.00194901 | up |
| Uric acid | 3.34361151 | 0.00101042 | up |
| Gibberellin A87 | 3.32479556 | 0.00520665 | up |
| Ornithine | 3.30909297 | 0.00011771 | up |
| (3-Methyl-2-butenyl)-benzene | 3.29532688 | 0.00037282 | up |
| N-Acetylneuraminic acid | 3.29453988 | 0.00189222 | up |
| 2-hydroxystearate | 3.19481355 | 0.00071937 | up |
| 2-Methyl-5-(8-pentadecenyl)-1,3-benzenediol | 3.19427113 | 0.00042759 | up |
| Procaine | 3.17602518 | 0.00432812 | up |
| N-Acetyldihydrosphingosine | 3.15132052 | 3.76E-05 | up |
| cis-Vaccenic acid | 3.09296899 | 5.34E-06 | up |
| Citalopram | 3.03436421 | 0.00198111 | up |
| Methyl .gamma.-linolenate | 2.96541766 | 0.00157869 | up |
| N4-Acetylsulfadimethoxine | 2.89566778 | 0.00196351 | up |
| 5Z,11Z,14Z-Eicosatrienoic acid | 2.86172876 | 0.00096857 | up |
| DL-Dopa | 2.85096781 | 0.00027943 | up |
| Ethylmalonic acid | 2.68311955 | 8.02E-05 | up |
| Benzeneethanamine, 2,5-dimethoxy-N-[(2-methoxyphenyl)methyl]-4-nitro- | 2.63982555 | 0.00098761 | up |
| Americanol A | 2.60757371 | 0.00203945 | up |
| 1H-Pyrano[3',4':6,7]indolizino[1,2-b]quinoline-3,14(4H,12H)-dione, 4,11-diethyl-4,9-dihydroxy-, (4S)- | 2.60422993 | 0.00383272 | up |
| Ile-Phe | 2.55850081 | 0.00090234 | up |
| 2-Acetamido-2-deoxy-3-O-(.beta.-D-galactopyranosyl)-D-glucopyranose | 2.55355217 | 0.00316977 | up |
| L-Fucose-1P | 2.5392007 | 0.00451055 | up |
| Gibberellin A87 | 2.47895188 | 0.00519219 | up |
| Cycloartocarpesin | 2.4779125 | 0.00218489 | up |
| 1,3,11-Tridecatriene-5,7,9-triyne | 2.47587441 | 0.00020074 | up |
| 3-Methoxy-2-(4-methylbenzoyl)-4H-1-benzopyran-4-one | 2.42758712 | 0.00098253 | up |
| cis-5,8,11,14-Eicosatetraenoic acid | 2.41536943 | 0.00839768 | up |
| Serine | 2.37122367 | 0.00677074 | up |
| Thiopental | 2.36686285 | 0.00024708 | up |
| 3-[3-Carboxy-2,3-dihydro-2-(4-hydroxy-3-methoxyphenyl)-7-methoxy-5-benzofuranyl]-2-propenoic acid | 2.33066476 | 0.00580557 | up |
| 5Z,11Z,14Z-Eicosatrienoic acid | 2.32084745 | 0.00599671 | up |
| 2-Deoxyribonic acid | 2.26928892 | 0.00060038 | up |
| (+)-2,3-Dihydro-3-methyl-1H-pyrrole | 2.26343906 | 0.00025679 | up |
| L-Serine | 2.1145607 | 0.00318819 | up |

Supplement table 4

The details list of significant upregulated Metabolites in C57BL/6J-5Gy compared with C57BL/6J -NC, ordered depending on FC. FC rounded 2 numbers after the decimal point. FC: Fold change.

| Metabolite | FC | t.test_p.value_BHcorrect | regulated |
| --- | --- | --- | --- |
| p-Cresol sulfate | 73.4817688 | 0.00080418 | up |
| Indoxyl sulfate | 62.9063689 | 0.00057119 | up |
| 3-Pyridinecarboxylic acid, 2-[(2-phenylethyl)thio]- | 32.3849264 | 0.000158 | up |
| 3-Hydroxybenzaldehyde | 30.4055886 | 2.18E-05 | up |
| Flufenacet ESA | 23.0580405 | 2.18E-05 | up |
| 2-Oxo-4-methylthiobutanoic acid | 18.5608754 | 0.00068261 | up |
| PG 14:0; PG(7:0/7:0) | 16.0835724 | 0.00018751 | up |
| 2-Methyl-5-(8-pentadecenyl)-1,3-benzenediol | 15.5021399 | 0.00100532 | up |
| Piperidine | 15.3823565 | 0.00208546 | up |
| 3-Coumaric acid | 13.9432526 | 9.67E-06 | up |
| N-Acetylvanilalanine | 13.66572 | 0.00136458 | up |
| Rivastigmine | 12.4768627 | 1.55E-05 | up |
| 4-Hydroxybenzaldehyde | 10.7569189 | 7.38E-05 | up |
| Phenol sulphate | 10.6909404 | 0.01170814 | up |
| 3a,6b,7b,12a-Tetrahydroxy-5b-cholanoic acid | 10.5707443 | 5.81E-05 | up |
| 3-Oxo-4,6-choladienoic acid | 10.4091632 | 7.44E-05 | up |
| (S)-2,3,4,5-tetrahydropyridine-2-carboxylate | 10.3519044 | 0.00410661 | up |
| xi-2,3-Dihydro-3-methylfuran | 10.266428 | 0.00094453 | up |
| Uric acid | 10.1803114 | 0.00016946 | up |
| Medicagenic acid | 9.78457773 | 9.59E-07 | up |
| 3-(2-Hydroxyphenyl)propionic acid | 9.60127854 | 0.00612594 | up |
| Cinobufagin | 8.69915433 | 0.0035918 | up |
| 17.alpha.-Dihydroequilin | 8.15175797 | 8.49E-10 | up |
| Methylglutaric acid | 7.94821086 | 0.00094974 | up |
| Docosapentaenoic acid | 7.88929918 | 3.70E-05 | up |
| 2-Butyl-3-methylpyrazine | 7.7852522 | 0.00306741 | up |
| ent-8(17),13(16),14-Labdatrien-18-oic acid | 7.2944385 | 6.66E-10 | up |
| beta-Zearalenol | 7.27702595 | 2.36E-06 | up |
| Methyl .gamma.-linolenate | 6.95870187 | 1.22E-06 | up |
| N-Arachidonoyl-3-hydroxy-.gamma.-aminobutyric acid | 6.75701674 | 0.00335498 | up |
| LysoPE 18:0 | 6.53662617 | 6.96E-06 | up |
| Exemestane | 6.17835179 | 0.00047373 | up |
| 4-Methylumbelliferyl heptanoate | 6.04644422 | 0.00052064 | up |
| 8Z,14Z-Eicosadienoic acid | 5.99680007 | 5.16E-05 | up |
| Arachidonic acid | 5.97583435 | 1.08E-07 | up |
| Methacrolein | 5.87875613 | 0.00057356 | up |
| 5,8,11-Eicosatrienoic acid | 5.8216892 | 0.00019238 | up |
| Stearic acid | 5.79700705 | 2.03E-08 | up |
| Pinolenic acid ethyl ester | 5.76130688 | 1.13E-10 | up |
| 2-Indolinone | 5.70340338 | 3.70E-05 | up |
| Physalin P | 5.50650415 | 0.0047297 | up |
| 3,7-Dihydroxy-12-oxocholanoic acid | 5.49036615 | 3.31E-05 | up |
| cis-4,7,10,13,16,19-Docosahexaenoic acid | 5.48737561 | 7.30E-09 | up |
| Lovastatin hydroxy acid | 5.46347881 | 2.58E-05 | up |
| Honyucitrin | 5.37854276 | 0.0053892 | up |
| 12-Ketodeoxycholic acid | 5.34382022 | 0.00275678 | up |
| O-Arachidonoylethanolamine | 5.28582909 | 4.39E-06 | up |
| 2-Methoxybenzoic acid | 5.2857989 | 0.00030393 | up |
| 12-oxo-20-dihydroxy-leukotriene B4 | 5.26325682 | 0.00051606 | up |
| Cavipetin C | 5.09055164 | 2.51E-07 | up |
| 3-(8,11,14-Pentadecatrienyl)phenol | 4.9839758 | 2.40E-06 | up |
| Procaine | 4.9704722 | 2.83E-08 | up |
| 8-iso-Prostaglandin A1 | 4.91392116 | 8.43E-06 | up |
| 7alpha-hydroxy-3-oxochol-4-en-24-oic Acid | 4.89092611 | 0.00115262 | up |
| 5Z,11Z,14Z-Eicosatrienoic acid | 4.77803702 | 4.93E-05 | up |
| cis-5,8,11,14-Eicosatetraenoic acid | 4.64908234 | 5.07E-06 | up |
| bicyclo-PGE2 | 4.47317411 | 0.00010571 | up |
| 2-Hydroxy-6-pentadecylbenzoic acid | 4.41928615 | 8.33E-06 | up |
| Stearoyl-L-carnitine | 4.39604015 | 3.48E-07 | up |
| L-Tyrosine | 4.38150286 | 1.79E-06 | up |
| Taurine | 4.37611637 | 0.00683925 | up |
| Phenol | 4.34147029 | 6.49E-05 | up |
| Necrofibrin, human | 4.23773791 | 0.00035123 | up |
| 2-hydroxystearate | 4.22475745 | 0.00010018 | up |
| Zeanic acid | 4.14597553 | 0.00119967 | up |
| 7a,12a-Dihydroxy-3-oxo-4-cholenoic acid | 4.10888137 | 0.00013469 | up |
| 3-Methyldioxyindole | 4.09295279 | 0.01227613 | up |
| cis-5,8,11,14,17-Eicosapentaenoic acid | 3.97022907 | 6.60E-05 | up |
| Prostaglandin H2 | 3.88411756 | 0.00017655 | up |
| 7-Ketodeoxycholic acid | 3.6858424 | 4.93E-11 | up |
| Estrone | 3.62973599 | 9.13E-06 | up |
| 2-Piperidinone | 3.60130698 | 0.00858316 | up |
| Ursocholic acid | 3.57366029 | 3.04E-05 | up |
| L-Carnitine | 3.53150892 | 0.00022199 | up |
| 13-cis Retinol | 3.4799677 | 3.76E-07 | up |
| Acylcarnitine 19:4 | 3.46089041 | 0.00034554 | up |
| 1,1'-[1,12-Dodecanediylbis(oxy)]bisbenzene | 3.44694475 | 0.00033344 | up |
| Resibufogenin | 3.42404892 | 2.75E-05 | up |
| Prostaglandin I3 | 3.3822637 | 0.00936251 | up |
| Arachidonoylglycine | 3.36983184 | 0.00220773 | up |
| Pentadecanoyl ethanolamide | 3.28585538 | 1.20E-07 | up |
| 1b,3a,12a-Trihydroxy-5b-cholanoic acid | 3.26556403 | 4.14E-05 | up |
| Isolithocholic acid | 3.21296204 | 0.0031993 | up |
| Anhydrocinnzeylanol | 3.18825405 | 1.31E-05 | up |
| Pilocarpine | 3.18610502 | 0.00313797 | up |
| 3-Acetyl-7-diethylaminocoumarin | 3.17805728 | 0.00506576 | up |
| 1a,11b-Dihydro-4,9-dimethylbenz[a]anthra[3,4-b]oxirene | 3.12561913 | 0.00339017 | up |
| LysoPC 16:0 | 3.10648175 | 0.00154913 | up |
| 9'-Carboxy-alpha-chromanol | 3.0977331 | 4.91E-05 | up |
| 6-beta-hydroxymedroxyprogesterone | 3.08194585 | 0.00479617 | up |
| Ganoderic acid A | 3.03494803 | 0.00050371 | up |
| Bufalin | 2.99158891 | 0.0002653 | up |
| Anileridine | 2.99116138 | 0.00017525 | up |
| 5a-Androst-3-en-17-one | 2.92227414 | 0.006166 | up |
| 4-Imidazoleacetic acid | 2.91821535 | 0.00074419 | up |
| Cardanoldiene | 2.88660162 | 0.00019517 | up |
| Xanthurenic acid | 2.83012874 | 0.00038757 | up |
| beta-Citraurin | 2.81542299 | 0.00267501 | up |
| Benzaldehyde | 2.78564474 | 0.0096372 | up |
| 16-Hydroxyhexadecanoic acid | 2.75785227 | 0.00272426 | up |
| Palmitoylcarnitine | 2.73678733 | 5.58E-05 | up |
| Docosahexaenoic acid methyl ester | 2.71541752 | 0.00053158 | up |
| Benzoylecgonine | 2.70681603 | 0.00465007 | up |
| Indole | 2.65406161 | 0.00123224 | up |
| 3-Formylindole | 2.64833564 | 0.00361906 | up |
| 3-Methyl-1-cyclohexanecarboxylic acid | 2.64362933 | 0.00981685 | up |
| Ricinoleic acid | 2.62209801 | 0.00146962 | up |
| Ortho-Hydroxyphenylacetic acid | 2.6188871 | 0.00812059 | up |
| Stachydrine | 2.60504772 | 0.00991215 | up |
| Gerberinol | 2.57546548 | 5.83E-05 | up |
| 3-Hydroxycinnamic acid | 2.56690884 | 0.00162314 | up |
| Methenolone | 2.54727958 | 1.55E-07 | up |
| Genistein | 2.54344195 | 0.00116459 | up |
| 3-Oxooctadecanoic acid | 2.54287406 | 0.00461886 | up |
| Hydroxygaleon | 2.53651367 | 5.84E-08 | up |
| Acylcarnitine 14:3 | 2.52429286 | 0.00841405 | up |
| 2-Phenylacetamide | 2.52143254 | 0.00096453 | up |
| 2-Linoleoylglycerol | 2.51966085 | 0.00078652 | up |
| Tyrosine | 2.51855875 | 0.00181208 | up |
| 13,14-Dihydro-15-ketoprostaglandin A2 | 2.50583512 | 0.00205786 | up |
| AMP | 2.47633492 | 0.00566181 | up |
| 4-Hydroxyquinoline | 2.44442071 | 0.00185489 | up |
| 11'-Carboxy-alpha-chromanol | 2.43882488 | 0.00051704 | up |
| Linoleic acid | 2.4285786 | 0.00185307 | up |
| Olmesartan medoxomil methyl ether | 2.39568307 | 0.00825655 | up |
| Perindopril | 2.38893632 | 0.00689971 | up |
| (25S)-7-Dafachronic acid | 2.38140096 | 0.00172083 | up |
| Citalopram | 2.29154293 | 0.0012302 | up |
| Nutriacholic acid | 2.26980067 | 0.00091264 | up |
| Choline | 2.26388271 | 0.00572036 | up |
| 4-Hydroxybenzoic acid | 2.24725507 | 0.00252833 | up |
| Acylcarnitine 20:6 | 2.23545353 | 0.00813572 | up |
| 1H-Indole-1-pentanoic acid, 3-[(4-chloro-1-naphthalenyl)carbonyl]- | 2.2347951 | 0.00260742 | up |
| 1-Methylguanine | 2.22481234 | 0.00485694 | up |
| 6,7-Dihydro-4-(hydroxymethyl)-2-(p-hydroxyphenethyl)-7-methyl-5H-2-pyrindinium | 2.22010726 | 2.04E-05 | up |
| 5,6-Epoxy-8Z,11Z,14Z-eicosatrienoic acid | 2.18581611 | 0.00056304 | up |
| Acylcarnitine 18:1 | 2.17935362 | 0.00897562 | up |
| Testosterone cypionate | 2.17854573 | 0.00388187 | up |
| 2-Acetamido-2-deoxy-3-O-(.beta.-D-galactopyranosyl)-D-glucopyranose | 2.08383379 | 1.57E-05 | up |
| Cadabicine | 2.07537859 | 0.00015357 | up |
| Cucurbitacin I | 2.05126608 | 0.00065594 | up |
| 3-Methoxy-2-(4-methylbenzoyl)-4H-1-benzopyran-4-one | 2.02908446 | 0.00777313 | up |
| 2-Oxopentanedioic acid | 2.02842759 | 0.00177192 | up |
| 11'-Carboxy-gamma-tocotrienol | 2.02735185 | 0.00485588 | up |
| 2b,3a,7a-Trihydroxy-5b-cholanoic acid | 2.01659599 | 0.00296803 | up |

Supplement table 5

The details list of significant upregulated Metabolites in both Balb/c-5Gy and C57/BL-6J-5Gy.

| 1 | 3-Hydroxybenzaldehyde |
| --- | --- |
| 2 | PG 14:0; PG(7:0/7:0) |
| 3 | 2-Methyl-5-(8-pentadecenyl)-1,3-benzenediol |
| 4 | Piperidine acid |
| 5 | 4-Hydroxybenzaldehyde |
| 6 | 3-Oxo-4,6-choladienoic acid |
| 7 | Uric acid |
| 8 | 17.alpha.-Dihydroequilin |
| 9 | Docosapentaenoic acid |
| 10 | beta-Zearalenol |
| 11 | Methyl .gamma.-linolenate |
| 12 | Exemestane |
| 13 | Methacrolein |
| 14 | 12-oxo-20-dihydroxy-leukotriene B4 |
| 15 | Procaine |
| 16 | 5Z,11Z,14Z-Eicosatrienoic acid |
| 17 | cis-5,8,11,14-Eicosatetraenoic acid |
| 18 | 2-hydroxystearate |
| 19 | Estrone |
| 20 | Prostaglandin I3 |
| 21 | Anhydrocinnzeylanol |
| 22 | 16-Hydroxyhexadecanoic acid |
| 23 | Ricinoleic acid |
| 24 | Ortho-Hydroxyphenylacetic acid |
| 25 | Gerberinol |
| 26 | 3-Oxooctadecanoic acid |
| 27 | Hydroxygaleon |
| 28 | Linoleic acid |
| 29 | Citalopram |
| 30 | 2-Acetamido-2-deoxy-3-O-(.beta.-D-galactopyranosyl)-D-glucopyranose |
| 31 | Cadabicine |
| 32 | 3-Methoxy-2-(4-methylbenzoyl)-4H-1-benzopyran-4-one |
